# The long-term gut bacterial signature of a wild primate is associated with a timing effect of pre- and postnatal maternal glucocorticoid levels

**DOI:** 10.1186/s40168-023-01596-w

**Published:** 2023-07-27

**Authors:** Simone Anzà, Dominik Schneider, Rolf Daniel, Michael Heistermann, Somboon Sangmaneedet, Julia Ostner, Oliver Schülke

**Affiliations:** 1 Behavioral Ecology Department, University of Goettingen, Goettingen, Germany; 2 Primate Social Evolution Group, German Primate Center, Leibniz Institute for Primate Research, Goettingen, Germany; 3 Leibniz ScienceCampus Primate Cognition, German Primate Center, Leibniz Institute for Primate Research, Goettingen, Germany; 4 Genomic and Applied Microbiology and Göttingen Genomics Laboratory, Institute of Microbiology and Genetics, University of Göttingen, Göttingen, Germany; 5 Endocrinology Laboratory, German Primate Center, Leibniz Institute for Primate Research, Goettingen, Germany; 6 Department of Pathobiology, Faculty of Veterinary Medicine, Khon Kaen University, Khon Kaen, Thailand

**Keywords:** Development, Prenatal stress, Dysbiosis, Macaques, Primates, Programming, Health, Long-term, Bacteria, 16S rRNA gene

## Abstract

**Background:**

During development, elevated levels of maternal glucocorticoids (GCs) can have detrimental effects on offspring morphology, cognition, and behavior as well as physiology and metabolism. Depending on the timing of exposure, such effects may vary in strength or even reverse in direction, may alleviate with age, or may concern more stable and long-term programming of phenotypic traits. Maternal effects on gut bacterial diversity, composition, and function, and the persistence of such effects into adulthood of long-lived model species in the natural habitats remain underexplored.

**Results:**

In a cross-sectional sample of infant, juvenile, and adult Assamese macaques, the timing of exposure to elevated maternal GCs during ontogeny was associated with the gut bacterial community of the offspring. Specifically, naturally varying maternal GC levels during early but not late gestation or lactation were associated with reduced bacterial richness. The overall effect of maternal GCs during early gestation on the gut bacterial composition and function exacerbated with offspring age and was 10 times stronger than the effect associated with exposure during late prenatal or postnatal periods. Instead, variation in maternal GCs during the late prenatal or postnatal period had less pronounced or less stable statistical effects and therefore a weaker effect on the entire bacterial community composition, particularly in adult individuals. Finally, higher early prenatal GCs were associated with an increase in the relative abundance of several potential pro-inflammatory bacteria and a decrease in the abundance of *Bifidobacterium* and other anti-inflammatory taxa, an effect that exacerbated with age.

**Conclusions:**

In primates, the gut microbiota can be shaped by developmental effects with strong timing effects on plasticity and potentially detrimental consequences for adult health. Together with results on other macaque species, this study suggests potential detrimental developmental effects similar to rapid inflammaging, suggesting that prenatal exposure to high maternal GC concentrations is a common cause underlying both phenomena. Our findings await confirmation by metagenomic functional and causal analyses and by longitudinal studies of long-lived, ecologically flexible primates in their natural habitat, including developmental effects that originate before birth.

Video Abstract

**Supplementary Information:**

The online version contains supplementary material available at 10.1186/s40168-023-01596-w.

## Background

Gestation is a developmental phase highly sensitive to environmental exposures; from conception onwards, adverse conditions and the resulting prenatal maternal stress response can affect offspring developmental trajectories depending on the type and degree of the adversity, the timing of such challenges, and offspring sex [1]. In mammals, maternal stress and increased activity of the hypothalamic–pituitary–adrenal (HPA) axis are associated with increased glucocorticoid (GC) production, which can permeate the placenta and reach the developing embryo/fetus during gestation. Prenatal maternal GCs are typically associated with long-lasting effects on morphological, cognitive, and physiological traits of the offspring [2, 3]. Specifically, phenotypic functional changes in physiological and behavioral traits associated with increased exposure to GCs during gestation involve not only the offspring’s HPA axis and immune system but also altered gene expression, behavior, and brain functionality [1, 4, 5]. An altered gut–brain system can be associated with detrimental variation of physiological traits such as impaired response to stressors and gut inflammation and altered behavior [6–15]. Yet, little is known about the programming effects of maternal stress on the gut microbiota, the possible mediating role of offspring GC levels, and the potential permanence of dysbiotic states into adulthood in long-lived mammals.

Starting from birth, the gut microbiota plays a fundamental role in regulating host physiology and behavior: it modulates metabolic and immune responses and affects brain development and adult behavior. Studies on germ-free animals show that bacteria-deprived individuals differ considerably in the development of physiological systems typical of individuals hosting microbial organisms such as impaired immune system, increased HPA axis activity, and altered metabolism [16] and emphasized the fundamental role of the gut microbiota in modulating the HPA axis activity and the stress response. One example is the modulation of the exaggerated HPA stress response in germ-free mice reduced by *Bifidobacterium infantis* colonization or increased by mono-association with enteropathogenic *Escherichia coli* [6]. Also in germ-free mice, *Enterococcus faecalis* reduced corticosterone levels and thus the physiological stress response following social stress [7]. However, studies on germ-free mice may overestimate stress-related physiological outcomes associated with variation in the abundance of single bacterial species, as isolation and lack of host–environment interactions may mask more complex microbial community dynamics. Thus, studies on animal models interacting with a rich microbial environment may help unveil hidden microbial dynamics and provide a deeper understanding of the link between stress-related physiology and the gut microbiota.

Stress can alter the secretions and permeability of the gastrointestinal tract, impact mucosal regenerative processes, and affect gastrointestinal motility [8]. Bailey and Coe [9] investigated stress-induced changes in the gut bacteria of rhesus macaques following maternal separation and showed that separation stress at 6–9 months of age caused a decrease in several taxa, particularly lactobacilli. The decrease was associated with stress-related behavior and with opportunistic bacterial infection and thus susceptibility to disease [9]. Indeed, the release of catecholamines produced during the stress response can stimulate the proliferation of gram-negative organisms [10] with direct or indirect effects on the abundance of taxa such as *Clostridium*, *Bacteroides*, and *Lactobacillus* [9]. A recent study of the effect of physiological stress associated with the process of habituation to humans in gorillas revealed the association of fecal glucocorticoid metabolites with increased abundance of the genus *Oscillobacter*, the *Clostridium* cluster XIV, and the family *Anaerolineaceae* [11]. However, despite changes in a few specific taxa, fecal glucocorticoid metabolites and bacterial alpha diversity were not associated indicating that stress from habituation had little effect on the overall gut microbial composition and more studies on the stress physiology of wild populations are needed [11].

Greater diversity and richness in the microbial community may provide greater resilience to perturbations and resistance to pathogens, improve the stability of the microbiota, and ultimately promote health. In humans, gut microbial richness correlates with metabolic markers and individuals with lower richness tend to have a pro-inflammatory phenotype, dyslipidemia, and insulin resistance [12]. The impoverishment of gut microbial alpha diversity may be associated with adverse health outcomes and altered behavior in human and non-human primates. For example, in mouse lemurs, rhesus macaques, chimpanzees, and humans, lower microbial richness is associated with infection by adenovirus, SIV, HIV, or enterocolitis [13–16]. Lower richness is also associated with stress, depressive-like, and anxiety-like behavior in humans and other primates [17–19], with Alzheimer’s disease in humans [20], and with helminth infections in yellow baboons and red colobus monkeys [21]. Interestingly, fecal microbiota transplantation from depressed patients (and with lower alpha-diversity) to microbiota-depleted rats induced alterations in tryptophan metabolism, depression-like, and anxiety-like behaviors in the recipient animals [20]. Despite growing evidence of a negative relationship between health and microbial diversity and richness, there are also several studies showing no association, and consequently, the debate on the use of diversity and richness as informative health markers is ongoing [11, 22, 23].

The gut microbiota seems very sensitive to stress, but the relationships between specific microbial taxa and host physiology are complex and tend to vary among host species, groups, and individuals. Nevertheless, some generalizations about the effect of specific taxa are possible. The phyla *Firmicutes* and *Bacteroidota* (formerly *Bacteroidetes*) represent two of the most abundant gut taxa in several mammalian species including humans, and their prevalence is influenced by environmental and genetic factors. Potentially, variation in the *Firmicutes*/*Bacteroidota* ratio may lead to dysbiotic states and metabolic dysfunction in humans and other primates as well [24–26]. This hypothesis is supported by rodent models, and studies on the effect of early-life stress on adults’ microbiota revealed a reduced *Firmicutes*/*Bacteroidota* ratio in adult females and an increased abundance of organisms associated with inflammation such as *Prevotella*, *Akkermansia,* and *Flexibacter* [27].

Among mammals, prenatal stress can strongly affect developmental trajectories of the brain function, HPA axis regulation, and other health-related traits [1, 3, 28, 29]. Consistently, in the study groups of wild Assamese macaques (*Macaca assamensis*), prenatal maternal stress during early, but not late gestation, or during lactation is associated with a hyperactivation of the HPA axis in the offspring, which persists into adulthood [30]. Indeed, the effects of prenatal maternal adversity and fetal exposure to GC on offspring phenotypes may have different, sometimes opposing, directions depending on the timing of the exposure—whether the exposure increases during the early or late gestation or after birth can be critical in shaping specific developmental trajectories and subsequent phenotypes [1, 31–33], with potential consequences for reproduction, health, and fitness [34, 35]. Studies on the effect of maternal stress on offspring gut microbiota are still limited and target a few host species, usually rodent models, in a controlled environment or in captivity [4, 36–38]. To date, we know of only one study that tested the timing effect of prenatal stress on offspring gut microbiota in a long-lived animal; this study on rhesus macaques was conducted in a controlled environment, using artificially induced stress and investigated effects only in infants [39]. Little is known about the ecological validity of such models, and even less is known about the potential persistence of maternal effects on adult gut microbiota in long-lived animal species [40].

With this study, we aim to provide data to fill this knowledge gap by investigating the link between the variation in maternal GCs driven by naturally occurring stressors, measured during different sensitive periods of gestation and lactation, and offspring gut microbial diversity, function, and composition in wild Assamese macaques. In a cross-sectional design during one field season (2018–2019), fecal samples were collected per individual and season from infants (< 1 year), juveniles (4–5 years), and adults (6–10 years) for the concurrent analysis of GC levels and gut bacterial community composition. Information on maternal GC levels during early and late gestation as well as during lactation was available from previous studies on the same population. We predicted increased prenatal and postnatal maternal GCs to be negatively associated with measures of microbial alpha diversity, the *Firmicutes*/*Bacteroidota* ratio, and with dysbiotic states evident from changes in the relative abundance of specific taxa and their predicted function. We further investigated whether these effects may be mediated by increased offspring GCs in developmentally challenged offspring by including offspring GC levels as a variable in all statistical models.

## Methods

### Study population and data collection

From 2010 to 2019, we collected fecal samples from a wild population of Assamese macaques living in their natural habitat. The study location is Phu Khieo Wildlife Sanctuary, a large, protected area that is part of a > 6500 km^2^ system of connected protected forests in northeastern Thailand. The study site (Huai Mai Sot Yai, 16° 27′ N, 101° 38′ E) is characterized by hilly terrain with a dry evergreen forest. The habitat has two distinct seasons: a hot and rainy season (rich: March–October) and a colder dry season (lean: November–February) [41–43]. Although fruit and food availability are higher during the rich season, resource availability fluctuates between years, and forest productivity is considered to be rather unpredictable [41–44].

The study population is mainly frugivorous, and the main part of the plant diet comprises fruit, pulp, and seeds. Individuals spend an important part of their feeding time slowly foraging for animal matter like insects, spiders, gastropods, small amphibians, and reptiles [41–43]. Assamese macaques are relaxed income breeders reproducing seasonally with 78% of births occurring between April and June (Schülke and Ostner unpublished data). Female reproduction depends on forest productivity and the probability of conception is positively associated with food availability and female condition [42]. The average length of gestation is estimated at 164 days [45]. Although infant suckling is observed through the first year of life, it is higher during the first 6 months and later it quickly decreases to zero [44]. Thus, we measured maternal postnatal GCs only for the first 6 months of life. All infant fecal samples used for DNA extraction and GC analysis were collected later, thus postnatal maternal GCs and infant offspring GCs were assessed at different times. Between 2010 and 2019, fecal samples from 30 mothers were collected during their gestation (*n* = 379, mean ± SD: 11.9 ± 5.7 per individual; preGC) and lactation (*n* = 564, 17.6 ± 6.2 per individual; postGC). Between June 2018 and July 2019, we collected fecal samples from the corresponding 30 offspring of different ages that lived in three different social groups: 231 (mean ± SD: 7.7 ± 2.3 per individual) samples were used to extract the information on GCs, and 217 (mean ± SD: 7.3 ± 1.9 per individual) were used to extract the information on gut bacterial communities. We measured fecal glucocorticoid metabolites and extracted information on the gut bacteria from offspring of the 2018 birth cohort (11 males and 5 females), juveniles born between 2014 and 2016 (4 males and 3 females), and individuals born before 2012 (adults, 2 males 5 females). Since the average length of gestation is 164 days, we divided gestation at day 82 into early gestation (gestation days 1–82) and late gestation (days 83–day of birth) and calculated specific means of GC concentration separately. We used two aliquots of fecal samples from offspring individuals to extract the information on GC metabolites and gut microbial community separately (see below).

### Fecal sample collection and glucocorticoid metabolite (GC) analysis

**Fig. 1:**
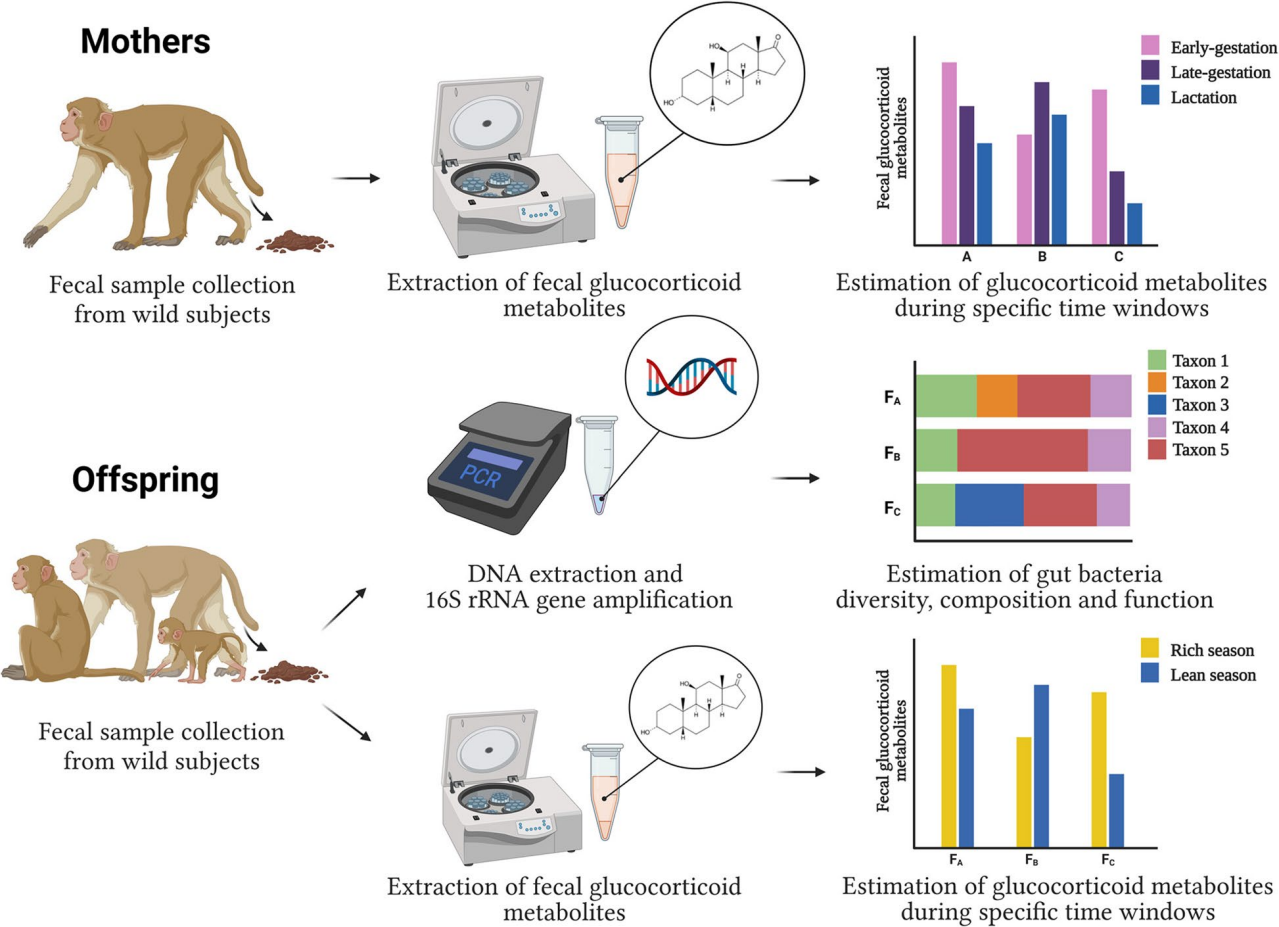
Methodological approach performed to investigate the effect of maternal GCs on offspring gut bacterial community and function. *F*_a-c_ refers to offspring descendent from mothers A–C of wild Assamese macaques. Output graphs are icons only and do not represent actual data. Created with Biorender.com

Fecal samples of mothers and offspring were collected directly after defecation (Fig. 1) from the ground or vegetation and without urine contamination. The fecal material was homogenized and about 1 g of material was transferred into a 15-ml vial with 5 ml 80% watery ethanol. The samples were extracted at the field camp using a validated method [46] as described by Berghänel et al. [44]. We pipetted 2 ml of the resulting fecal extract containing the GCs into a polypropylene cup and successively stored all extracts at −20 °C until transportation to the endocrinology lab of the German Primate Center for GC metabolite analysis. The extracts were analyzed using an enzyme immunoassay for the measurement of immunoreactive 11ß-hydroxyetiocholanolone, a major metabolite of GCs in primate feces [47]. The assay has been validated for assessing adrenocortical activity in numerous primate species [47], including Assamese macaques [48, 49]. Extracts were diluted 1:200–1:4,000 with assay buffer before assays which were then carried out according to the method described in detail in Heistermann et al. [50]. Assay sensitivity was 12 pg/ml, and intra- and inter-assay coefficients of variation of high- and low-value quality controls were < 10% and < 15%, respectively.

### Fecal sample collection, DNA extraction, amplification of 16S rRNA genes, and sequencing

We collected fecal samples of offspring directly after defecation (Fig. 1). We homogenized the samples by kneading them with gloved hands and put them in sterile 2-ml screw-top polypropylene cups filled with 1 ml RNAlater buffer solution, shook the samples, and then stored them for 24 h in the dark at room temperature. Subsequently, the samples were frozen at −20 °C until export to the Göttingen Genomics Laboratory at the University of Göttingen. Once in the lab, the samples were stored at −80 °C.

Samples stored at −80 °C were thawed on ice for 30 min. RNAlater was removed by centrifuging samples for 10 min at 13,000 rpm on a Thermo Electron Corp Heraeus Pico 21 (ThermoFisher Scientific). DNA was extracted from 150 mg of fecal matter with DNeasy PowerSoil Pro Kit (QIAGEN, Cat. No./ID: 47016) following manufacturer instructions. DNA quantity and quality were assessed by spectrophotometry on a NanoDrop ND-1000 Spectrophotometer (Thermo Fisher Scientific). Samples yielding a DNA concentration < 6 ng/µl were discarded and DNA extraction was repeated. We standardized DNA concentration per sample by dilution to 10 ng/µl. Using PCR primers as described by Klindworth and colleagues [51] we amplified the V3–V4 region of the 16S rRNA gene. Primers included adapters for MiSeq sequencing (underlined, forward primer: S-D-Bact-0341-b-S-17 5′-TCGTCGGCAGCGTCAGATGTGTATAAGAGACAG-CCTACGGGNGGCWGCAG-3′, reverse primer: S-D-Bact-0785-a-A-21 5′-GTCTCGTGGGCTCGGAGATGTGTATAAGAGACAG-GACTACHVGGGTATCTAATCC-3′). PCRs were performed in triplicates with thermocycling protocols listed in Supplementary material (SI Amplification of 16S rRNA genes and sequencing: full procedure). Sequencing was conducted by the Göttingen Genomics Laboratory using Illumina MiSeq platform using dual indexing and MiSeq reagent kit v3 (600 cycles) as recommended by the manufacturer (SI Amplification of 16S rRNA genes and sequencing: full procedure).

### 16S rRNA gene sequence data deposition

The raw sequence data from the 16S rRNA gene amplicons were deposited at the National Center for Biotechnology Information and can be assessed under the BioProject accession number PRJNA795139.

### Bioinformatic processing of amplicon data

Raw paired-end sequences were quality-filtered using fastp v0.20.0 [52] with a minimum phred score of 20, minimum sequence length of 50 bp and sliding window size of 4, read correction by overlap, and adapter removal of sequencing primers. Quality-filtered reads were merged with PEAR v0.9.11 [53], and 16S rRNA gene primers were trimmed with cutadapt v2.5 [54]. VSEARCH v2.15.0 [55] was used to sort and filter the sequences by size (allowed minimum length ≥ 300 bp), remove duplicates (--derep_fulllength), and denoise (--cluster_unoise, default settings). We further performed de novo chimera removal (--uchime3_denovo) followed by reference-based chimera removal (--uchime_ref) against the SILVA SSU 138.1 NR database [56] resulting the final set of Amplicon Sequence Variants (ASVs). Finally, merged and quality filtered sequences were mapped against ASVs with VSEARCH (--usearch_global) with default sequence identity threshold of 0.97. The bacterial lineage of each ASV were assigned by using BLASTn v2.9.0 + against SILVA SSU 138.1 NR [56]. Best hits were only accepted if $\left(\frac{\%\mathrm{identity}+\%\mathrm{coverage}}2\right)$ ≥ 93 following the recommendation of SILVA team [56]. A total of 25,283,812 reads corresponding to 3934 ASVs were obtained from the 411 samples, with all samples achieving high read counts (mean ± SD = 61,517 ± 31,959 reads per sample, median = 54,126, range = 21,010–262,994).

### Statistical analyses

To investigate the link between maternal GCs and potential detrimental dysbiosis in offspring’s gut bacteria, we began by exploring variation in microbial alpha diversity linked with prenatal maternal GCs during early and late gestation, postnatal maternal GCs, and associated with offspring GCs, sex, age, group, and season of data collection. The study subjects experienced the same environmental conditions in terms of food availability and climate during data collection. All analyses were performed in R Studio (Version 3.6.1) using the packages *glmmTMB* [57] and *ancombc* [58]. We ran GLMMs with a full-null model comparison to investigate several alpha diversity measures: pure richness-based estimators (ObservedASVs, ACE; Model 1a-1b), Faith’s phylogenetic diversity (PD) as phylogenetic richness estimator (Model 1c), and richness-evenness estimators (i.e., Shannon-Weaver, Inverse Simpson: Model 1d-1e). We ran all the models always including the same predictors: the average value of maternal GCs during early gestation (*Early-preGC*) and late gestation (*Late-preGC*), the average value of postnatal maternal GCs (*PostGC*), the average value of offspring GCs estimated separately for the rich and the lean season (*OffspringGC*), and the information on offspring sex, age category (infant, juvenile, adult), group (MST, MOT, SST), and season of data collection (rich, lean). We included the random intercept individual ID and the random slopes of *Early-preGC*, *Late-preGC*, *PostGC*, and *OffspringGC* within individual ID in all models. Because the inclusion of the random slopes of *Early-preGC*, *Late-preGC*, *PostGC*, and *OffspringGC* within individual ID caused no convergence of all full models, we removed the random slopes and re-ran reduced models. We removed all not significant interactions and included main terms in reduced models. All the covariates were log_n_-transformed and then z-transformed to achieve model requirements and improve the interpretability of predictors. We checked model stability and collinearity of predictors using the package *performance* which identified no collinear predictors. Each alpha diversity model dataset included 192 data points belonging to 30 subjects (mean = 6.4, SD = 2.07).

Successively, we performed the analysis of compositions with bias correction ANCOM-BC [58], which estimates the unknown sampling fractions and corrects the bias induced by their differences among samples. The absolute abundance data were modeled by ANCOM-BC using a linear regression framework. ANCOM-BC provides a statistically valid test with appropriate *p*-values, confidence intervals for differential abundance of each taxon, and controls the false discovery rate while maintaining adequate power in a computationally simple way of implementing the models. In total, we ran six separate ANCOM-BC analyses at phylum, family, and genus level for rich and lean season. We conducted separate analyses for the rich and the lean season since *ancombc* does not allow longitudinal analysis.

Before running ANCOM-BC, we merged the information on differential abundance derived from seasonal biological replicates of each subject into a single mean value per individual and season (rich season: 3.7 ± 1.0; lean season: 4.5 ± 1.5 samples). The predictors included in each ANCOM-BC model were always coded in the same way as the alpha diversity models except *Age* that was included as a continuous variable and z-transformed with mean = 0 and sd = 1 to avoid convergence issues and improve interpretation of each interaction term. We excluded the collinear predictor *Group* (VIF > 5). *p*-values were adjusted to control for false discovery rate [59] (Benjamini–Hochberg corrected *p*-values: *p*_Bh_ < 0.05). The model datasets on ANCOM-BC during the rich and lean seasons included 16 and 30 data points, respectively, because some individuals could not be adequately sampled in the rich season. To understand whether the effect of maternal and offspring GCs on gut microbial composition was constant across different seasons, we investigated the number of taxa significantly affected by the same model predictors during the rich and the lean season separately. Thus, we analyzed whether the effect direction of each predictor on different taxa was concordant or discordant between seasons.

We performed general linear models on the *Firmicutes* to *Bacteroidota* ratio (*Firmicutes*/*Bacteroidota*) using the same model predictors to investigate dysbiosis associated with maternal and offspring GCs moderated by age. We conducted a likelihood ratio test and obtained the *p*-values for each predictor variable using single-term elimination with the drop1 function in R [60]. The model datasets on *Firmicutes*/*Bacteroidota* during the rich and the lean seasons included 16 and 30 data points, respectively.

### Functional profiling of gut bacteria

Bacterial genes were inferred from the 16S rRNA gene derived ASVs using PICRUSt2 (Phylogenetic Investigation of Communities by Reconstruction of Unobserved States) v2.5.1 [61]. We followed the standard PICRUSt2 protocols (GitHub page: https://github.com/picrust/picrust2/wiki) to investigate the predicted gene families using the Kyoto Encyclopedia of Genes and Genomes (KEGG) Orthology (KO) database. PICRUSt2 prediction accuracy was assessed by calculating the weighted nearest sequence taxon index score (mean of NSTI = 0.133). We tested the effects of prenatal and postnatal maternal GCs in interaction with offspring age on the relative abundance of estimated functional KO categories grouped at level 3 of the KEGG pathway database using ANCOM-BC via the same methodological approach performed to investigate the gut bacterial community (see above).

## Results

### General gut bacterial community composition of free-living *Macaca assamensis*

**Fig. 2:**
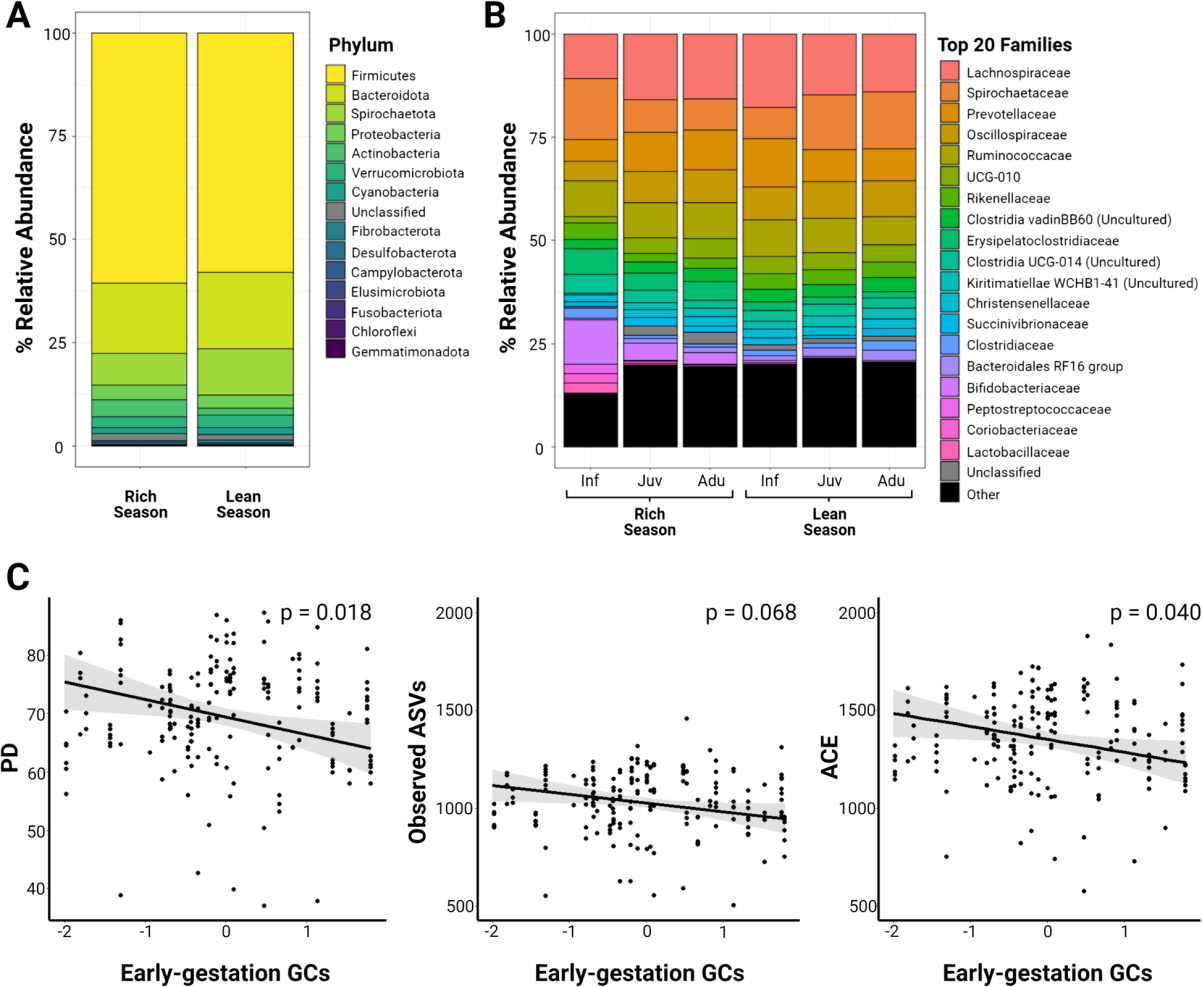
Gut bacterial community and effect of maternal GCs during the first half of gestation (*Early-preGC*) on alpha diversity measures in Assamese macaques during the study period, June 2018 to June 2019. Panel **A** shows the relative abundance of all bacterial phyla averaged by *Season*. Panel **B** shows the relative abundance according to *Age* and *Season* of the 20 most abundant families which accounted for 84.2% of the total composition. Panel **C** shows the effect of *Early-preGC* (z-transformed with mean = 0 and SD = 1) on Faith’s phylogenetic diversity (PD), observed ASVs, and abundance-based coverage estimator (ACE) indices. Each model line is plotted with all the other model predictors at their average value. All panels were plotted with R and converted with Biorender.com to be consistent within the text

The overall gut bacterial community composition was analyzed for the study population from 320 fecal samples collected from 51 subjects (6.3 ± 2.6 samples per subject), not all of whom had data on maternal GCs during development. Approximately 97% of the microbial composition was represented by seven bacterial phyla with similar composition during the rich and the lean seasons (Fig. 2A). Overall, the phylum *Firmicutes* was the most abundant one (59.3 ± 7.1%), followed by *Bacteroidota* (17.9 ± 5.2%), *Spirochaetota* (9.3 ± 6.5%), *Proteobacteria* (3.5 ± 3.2%), *Verrucomicrobiota* (2.9 ± 1.9%), *Actinobacteriota* (2.9 ± 5.3%), and *Cyanobacteria* (1.6 ± 1.1%). Together, the phyla *Fibrobacterota* (0.5 ± 0.7%), *Desulfobacterota* (0.4 ± 1.5%), *Campylobacterota* (0.3 ± 0.3%), and *Elusimicrobiota* (0.1 ± 0.4%) represented the remaining 3% (including 1.4% of unclassified bacteria).

At the family level, almost 80% of the overall main composition was represented by 15 families (~ 85% by 20 families): *Lachnospiraceae* (17.19 ± 6.2%) was the most abundant family, followed by *Prevotellaceae* (10.1 ± 5.7%), *Spirochaetaceae* (9.2 ± 6.6%), *Ruminococcaceae* (8.6 ± 3.1%), *Oscillospiraceae* (7.7 ± 2.8%), UCG*-*010 of the order *Oscillospirales* (3.9 ± 1.8%), *Rikenellaceae* (3.2 ± 1.9%), uncultured bacteria from the Clostridia vadin BB60 group (2.9 ± 1.6%), *Erysipelatoclostridiaceae* (2.8 ± 2.3%), uncultured bacteria from the Clostridia UCG-014 (2.4 ± 1.4%), *Christensenellaceae* (2.1 ± 1.0%), uncultured bacteria from the order WCHB1-41 belonging to the class of *Kiritimatiellae* (2.1 ± 1.6%), *Succinivibrionaceae* (1.8 ± 3.1%), *Butyricicoccaceae* (1.7 ± 1.5%), uncultured bacteria from the Coprostanoligenes group of *Oscillospirales* (1.7 ± 0.8%), and finally *Muribaculaceae* (1.6 ± 1.0%) (Fig. 2B).

### Maternal GCs during early gestation alter offspring’s bacterial diversity

Next, samples were filtered and only those belonging to individuals with complete information on maternal GCs during (early and late) gestation and lactation were retained. Thus, we estimated gut microbial alpha diversity from 192 fecal samples belonging to 30 subjects (6.4 ± 2.07 samples per subject). Overall, all full-models of alpha diversity consistently revealed no interaction effect of age with maternal GCs and with offspring GCs (Supplementary Tables 1 and 2). Therefore, we ran reduced models without non-significant interaction terms (Supplementary Table 3). Reduced models 1a-1c showed consistent results, and all predictors together clearly influenced the offspring’s gut bacteria alpha diversity in all models (Model 1a: *χ*2 = 72.85, *df* = 11, *p* < 0.001; Model 1b: *χ*2 = 91.98, *df* = 11, *p* < 0.001; Model 1c: *χ*2 = 66.38, *df* = 11, *p* < 0.001; Table 1, Supplementary Table 3). Increasing prenatal maternal GCs during early but not late gestation was significantly associated (or in one case tended to be) with a decrease in alpha diversity (Early-preGC: *p*_1a_ = 0.068, *p*_1b_ = 0.040, *p*_1c_ = 0.018, Fig. 2C; Late-preGC: *p*_1a_ = 0.503, *p*_1b_ = 0.670, *p*_1c_ = 0.999). Postnatal maternal GCs had a positive effect on alpha diversity, although only two models revealed a statistical trend (PostGC: *p*_1a_ = 0.089, *p*_1b_ = 0.054), and one revealed a non-significant effect on phylogenetic diversity (PostGC: *p*_1c_ = 0.148). Surprisingly, the seasonal offspring GCs mean value had no effect on the model responses (OffspringGC*: p*_1a_ = 0.395, *p*_1b_ = 0.313, *p*_1c_ = 0.987). Very consistently, Age, Season, and Group had the same significant effect in all alpha diversity models, while Sex had no effect on any response (Supplementary Table 3).

**Table 1:** Effects of model predictors on Faith’s phylogenetic diversity of offspring’s gut bacteria

**Model 1c – Faith’s phylogenetic diversity (*R***^**2**^** = 0.29)**					
**Predictors**	**Estimates**	**SE**	**CI 2.5–97.5%**	***Z***	***p*-value**
(Intercept)	68.21	2.01	64.26 to 72.15	33.88	**–**
Early-preGC	−2.09	0.88	−3.82 to −0.36	−2.37	**0.018**
Late-preGC	0.00	0.85	−1.67 to 1.67	0.00	0.999
PostGC	1.24	0.86	−0.44 to 2.93	1.45	0.148
OffspringGC	−0.01	0.77	−1.52 to 1.50	−0.02	0.987
^a^Sex [M]	0.20	1.30	−2.34 to 2.74	0.15	0.879
^b^Age [adu]	8.62	1.86	4.99 to 12.26	4.65	**< 0.001**
^b^Age [juv]	11.66	2.33	7.08 to 16.23	4.99	**< 0.001**
^c^Season [Rich]	−7.00	1.45	−9.85 to -4.15	−4.82	**< 0.001**
^d^Group [2]	−3.11	1.64	−6.32 to 0.10	−1.90	0.058
^d^Group [3]	−4.51	1.89	−8.21 to −0.81	−2.39	**0.017**

Reduced model 1c explaining the Faith’s phylogenetic diversity (PD) of offspring’s gut bacteria. Significant *p*-values of explanatory variables are in italics. *R*^2^ indicates the conditional coefficient of determination. All covariates are log_n_-transformed and then z-transformed (mean = 0, SD = 1) to meet model requirements and increase model interpretability

^a^Coded with “female” as the reference category

^b^Coded with “infant” as the reference category

^c^Coded with “lean” as reference category

^d^Coded with “1” as reference category

The models analyzing the richness-evenness estimators (Model 1d-1e) showed different results (Supplementary Table 4). Although the reduced-null model comparison revealed that all predictors together influenced the alpha diversity of offspring gut bacteria (reduced-Null model comparison of Model 1d: *χ*2 = 29.26, *df* = 15, *p* = 0.015; Model 1e: *χ*2 = 20.08, *df* = 11, *p* = 0.044), only *Season* and *Group* had a significant effect on the Shannon index (Model 1d), while only *Age[juv]* and *Group* significantly affected the Inverse Simpson index (Model 1e). In both Model 1d and 1e, prenatal maternal GCs during early and late gestation and postnatal maternal GCs had no statistically significant effect on richness-evenness estimators (Supplementary Table 4).

### Maternal GCs, offspring GCs, and overall variation in bacterial composition

The analysis of gut bacterial composition (ANCOM-BC) identified differentially abundant taxa according to predictors of interest such as *Early-preGC, Late-preGC, PostGC, OffspringGC, Age,* and *Sex* and their interactions. If a predictor variable was part of a significant interaction term, we do not report the summary statistics used to estimate variation in overall composition due to very limited interpretability. We provide a quantitative interpretation of predictor effects based on the percentage of taxa significantly affected, with the assumption that, regardless of effect size, the greater the number of taxa significantly affected, the greater the potential relevance of the model predictor in shaping the overall gut bacterial community composition.

#### Rich season

**Fig. 3:**
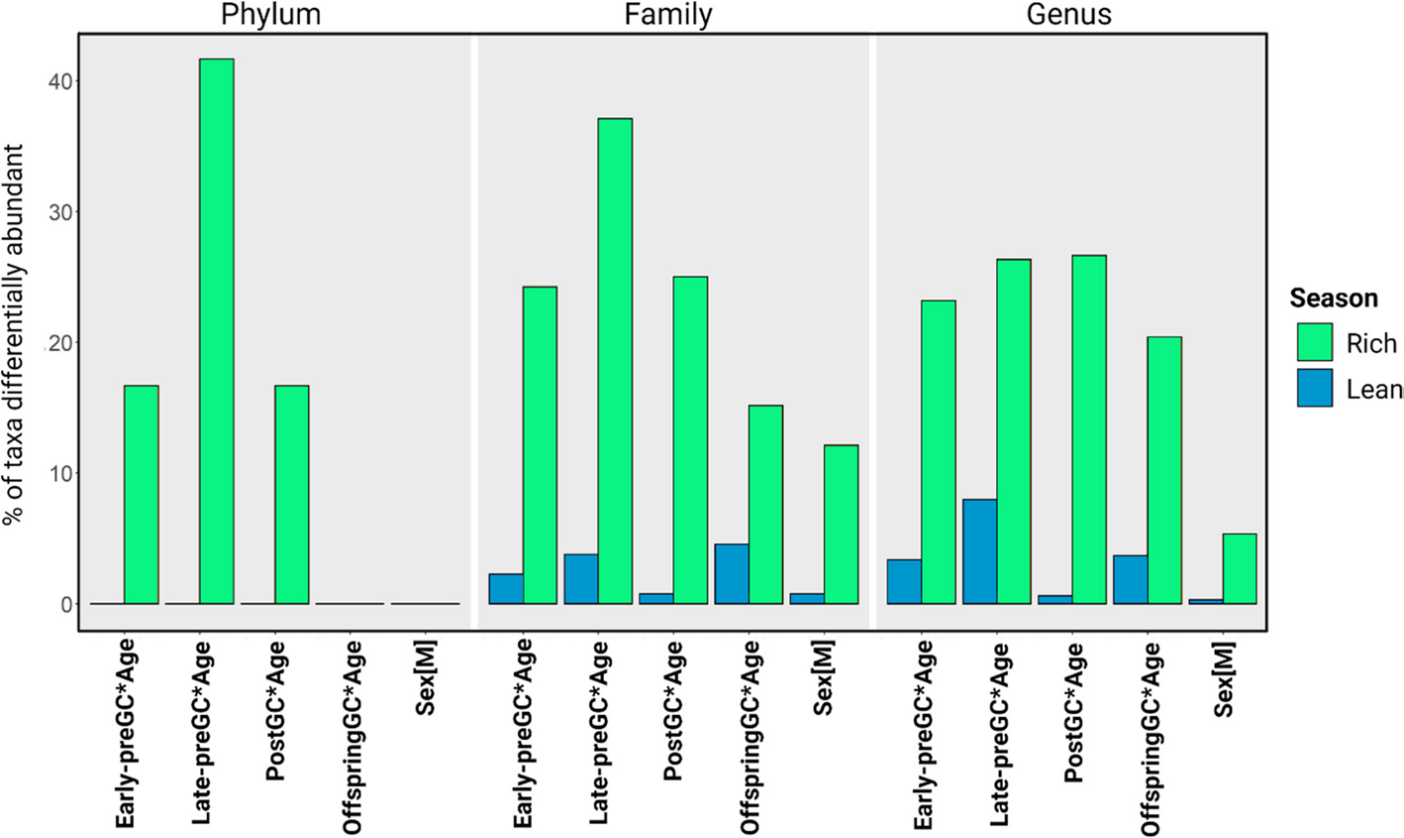
Percentage of bacterial taxa differentially abundant according to model predictors moderated by offspring’s age. The composition is estimated for the rich and the lean season at the phylum, family, and genus level separately. The significance of the main terms of each interaction has limited interpretability and therefore they are not depicted. All the variables but sex have been z-transformed with mean = 0 and SD = 1 to increase model interpretability. Sex is coded with “female” as the reference category. Early-preGC = prenatal maternal GCs during early gestation, Late-preGC = prenatal maternal GCs during late gestation, PostGC = postnatal maternal GCs during lactation, OffspringGC = seasonal offspring GCs. The figure has been plotted with R and edited with Biorender

At the phylum level, early prenatal maternal GCs and postnatal maternal GCs in interaction with age were associated with moderate differences whereas late prenatal GCs in interaction with age had considerable effects on bacterial composition: 1 SD of variation in maternal GCs with 1 SD of variation in age was associated with significant changes in the abundance of 10–20% and 20–40% of phyla, respectively. Offspring GC or sex in interaction with age did not affect gut bacterial composition in relevant ways. At the family and genus level, variation in maternal GCs during all three time-windows in interaction with age had considerable effects on the bacterial composition, and offspring GC effects increased to a moderate level (Fig. 3, Table 2).

**Table 2:** Quantitative characterization of model predictors’ relevance

**% of taxa significantly affected**	**Variation in the overall composition**	**Relevance**
**0–5**	No variation/similar composition	Not relevant
**5–10**	Minor differences	Slightly relevant
**10–20**	Moderate differences	Mildly relevant
**20–40**	Considerable differences	Considerably relevant
**> 40**	High differences	Highly relevant

Ranges of the percentage of taxa significantly affected by predictors used to quantitatively characterize the relevance of model predictors in shaping variation in overall microbial composition

#### Lean season

Overall, the gut bacterial composition during the lean season was more anchored and less influenced by predictors. Minor variation in composition was observed at the family and genus level in response to an interaction of age with late preGCs and offspring GC (Fig. 3).

### Bacterial composition and the effect of maternal GCs, offspring GCs, and age

Subsequent analyses focused on the direction of effects associated with prenatal and postnatal maternal GCs and offspring GCs in interaction with age. We report summary statistics on the modeled effect of maternal and offspring GCs at the mean of the variable age (equivalent to juveniles age class), mean + 1 SD (equivalent to adult age class), and mean-1 SD (roughly equivalent to infant age class). Exposure to maternal GCs before and after birth had significant effects on bacterial community composition depending on the age of the offspring and sampling season, but these effects during the lean season concerned less than 5% of taxa only (with one exception) and were typically less stable in the direction across age compared to effects during the rich season.

#### Rich season

**Fig. 4:**
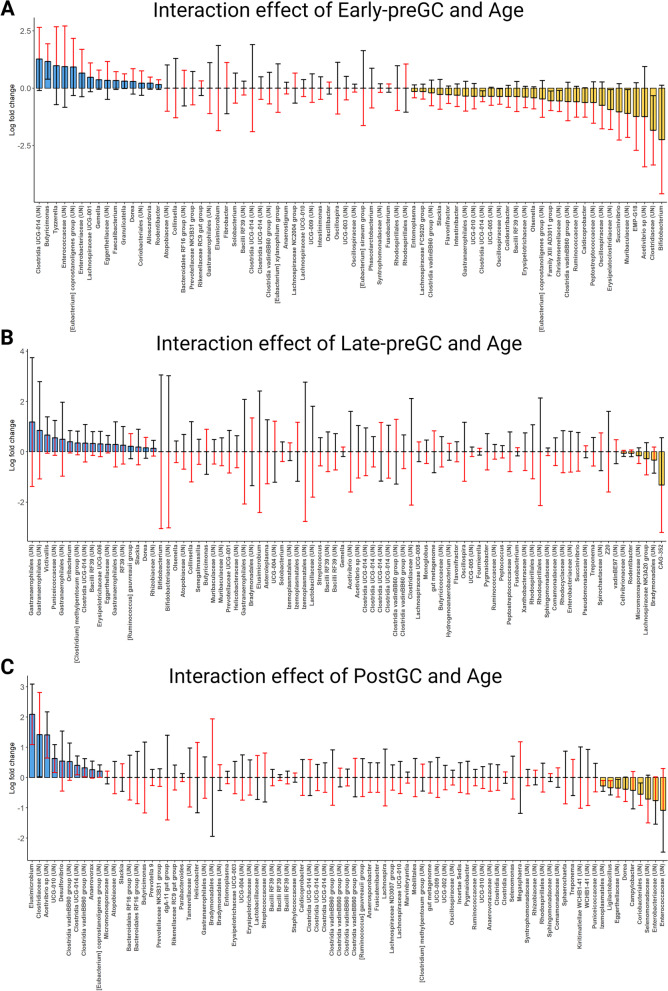
Effect of different maternal predictors moderated by *Age* at sampling on the differential relative abundance of bacterial genera during the rich season. Panel **A** shows the effect of *Early-preGC* moderated by *Age* (Early-preGC*Age), panel **B** shows the effect of *Late-preGC* moderated by *Age* (Late-preGC*Age), and panel **C** shows the effect of *PostGC* moderated by *Age* (PostGC*Age). “UN” refers to undetermined/unclassified genus, and family or order is therefore reported. Colored bars indicate the effect direction (blue = positive, yellow = negative) of an increase in 1 SD of specific predictors (*Early-preGC* = 59.3 ng/g; *Late-preGC* = 67.0 ng/g; *PostGC* = 52.7 ng/g) estimated at the mean value of age (mean = 4.7 years), and at the mean value of all the other predictors. Red and black lines indicate the effect estimated at the mean plus 1 SD (red = 7.1 years) and at the mean minus 1 SD (black = 2.3) of age. Case example: Panel **A** 1st taxon from the right: when subjects are 4.7 years old (mean age), the abundance of *Bifidobacterium* experiences a 2.3 log-fold decrease with an increase in maternal GCs during the early gestation of 59 ng/g (1SD). When the subjects are 2.3 years old (mean age-1SD), the same increase in maternal GCs is associated with an 0.12 log fold increase in *Bifidobacterium* abundance (black whisker). Finally, when the subjects are 7.1 years old (mean age + 1 SD), the same increase in maternal GCs is associated with a 4.6 log-fold decrease in the abundance (red whisker). When the bar is not plotted, the effect of the same increase in maternal GCs at the mean age is 0. Taxa showing the black head (estimates at 2.3 years) further from 0 than the red head (estimates at 7.1 years) show an effect that is reduced with age

Out of a total of 319 genera analyzed, 23.2% (74) were significantly affected by maternal GCs during early gestation moderated by age (Early-preGC*Age). The effect of a 1 SD increase in *Early-preGC* on differential abundance amplified with *Age* in 14.1% of the genera (positive: 4.7%; negative 9.4%), decreased with *Age* in 0%, and reversed in 9.1%. Late prenatal maternal GCs moderated by age (Late-preGC*Age) had a similar effect on microbial composition in terms of the number of genera significantly affected (84 out of 319, 26.3%). However, the effect of an increase of 1 SD of *Late-preGC* amplified with *Age* in only 1.6% of genera (positive: 0.6%; negative 1%), reduced in 3.8% (positive: 1.0%; negative: 2.8%), and reversed in as many as 20.9% of genera. A total of 85 out of 319 genera (26.6%) were affected by postnatal maternal GC moderated by age (PostGC*Age). The effect of *PostGC* amplified with *Age* in 1.9% of genera (positive: 0.3%; negative: 1.6%), weakened with *Age* in 4.1% (positive: 2.5%; negative: 1.6%) and mainly reversed with age in 20.6% of genera (Fig. 4). The ANCOM-BC analysis was repeated including only juveniles and adults and showed a similar effect of early-preGC on these genera (Supplementary Table 6).

**Fig. 5:**
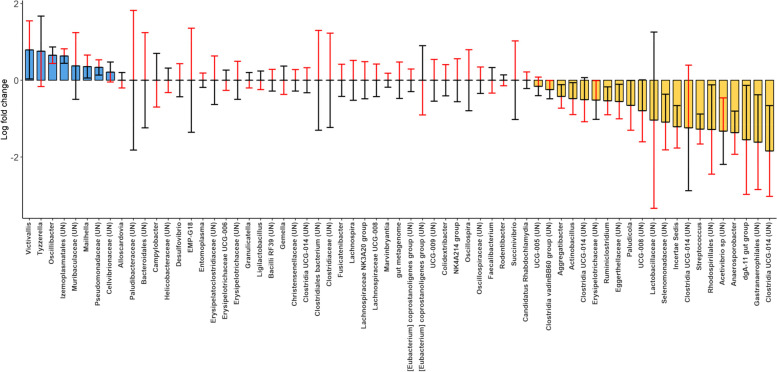
Effect of concurrent *OffspringGC* moderated by *Age* (OffspringGC*Age) on the differential abundance of genera during the rich season. “UN” refers to undetermined genus, and family or order is therefore reported. Colored bars indicate the effect (blue = positive, yellow = negative) of an increase in 1SD of *OffspringGC* (SD = 130.1 ng/g) estimated at the mean value of age (mean = 4.7 years), and at the mean value of all the other predictors. Red and black lines indicate the effect of *Late-preGC* estimated at the mean + 1 SD (red = 7.1 years) and at the mean – 1 SD (black = 2.3 years) of age

Finally, 65 out of 319 genera (20.4%) were affected by offspring GCs moderated by age (OffspringGC*Age). The effect of *OffspringGC* amplified with *Age* in 6% of genera (positive: 1.3%; negative: 4.7%), reduced in 2.2% (positive: 0.9%; negative: 1.3%), and reversed with *Age* in 12.2% (Fig. 5, Table 3).

**Table 3:** Effect of maternal and offspring GCs on gut bacterial composition

**Predictor**	**Taxa significantly affected**	**Amplify with age (%)**	**Attenuate with age (%)**	**Reverse with age (%)**
Early-preGC	74	60.8	0.0	39.2
Late-preGC	84	6.0	21.4	72.6
PostGC	85	5.9	16.5	77.6
OffspringGC	65	32.3	12.3	53.8

Effect of maternal and offspring GCs moderated by age on the gut bacterial composition at the genus level during the rich season. The direction of moderation (amplification, attenuation, reversal) is summarized for significantly affected genera only

#### Lean season

Out of a total of 326 genera analyzed, only 11 were significantly affected by maternal GCs during early gestation moderated by age (Early-preGC*Age), all of which reversed with age. Late prenatal maternal GCs moderated by age (Late-preGC*Age) had a similarly small effect on bacterial composition in terms of number of genera significantly affected (26 out of 326). The effect of *Late-preGC* amplified with *Age* in 2 genera (both positive), reduced with *Age* in 1 taxon (negative), and reversed with *Age* in 11 genera. Postnatal maternal GCs moderated by age (PostGC*Age) had a neglectable effect on bacterial composition (2 out of 326 genera), and only 12 genera were affected by offspring GC moderated by age (OffspringGC*Age). The effect of *OffspringGC* amplified with *Age* in 1 taxon (positive), attenuated in 1 taxon (negative), and reversed with *Age* in 10 genera (Fig. 5).

#### The *Firmicutes* to *Bacteroidota* ratio

**Fig. 6:**
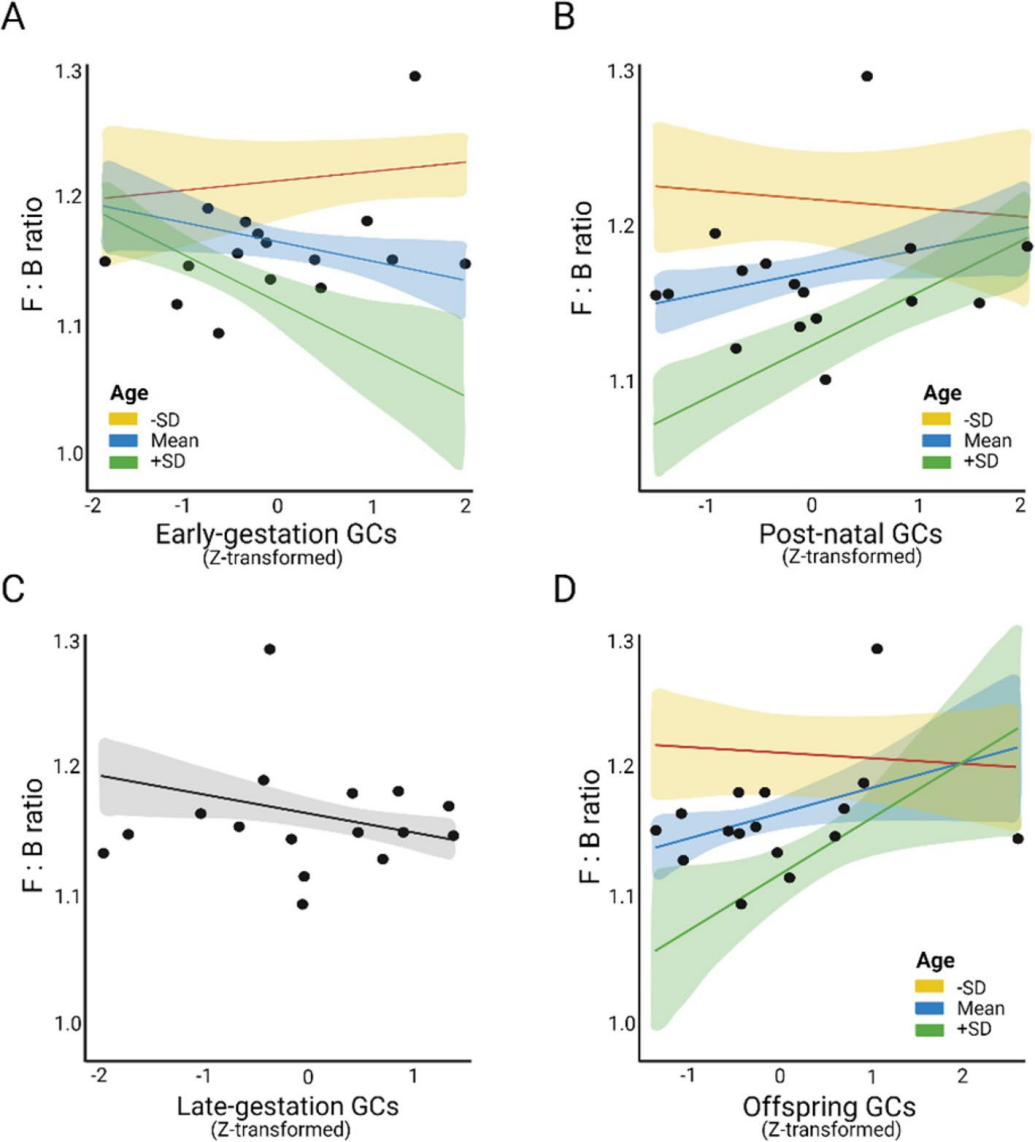
Effect of significant predictors of reduced Model R explaining variation in the *Firmicutes* to *Bacteroidota* ratio (F:B) of offspring’s gut bacteria during the rich season. Panel **A** shows the effect of prenatal maternal GCs during the first half of gestation moderated by offspring’s age (Early-preGC*Age). Panel **B** shows the effect of postnatal maternal GCs moderated by offspring’s age (PostGC*Age). Panel **C** shows the effect of prenatal maternal GCs during the second half of gestation (*Late-preGC)*. Panel **D** shows the effect of offspring GCs moderated by offspring’s age (OffspringGC*Age). The moderator *Age* is plotted at its mean and ± SD values (4.7 ± 2.4). Results on predictors shown in panels **A** and **C** corroborate the overall negative effect of prenatal maternal GCs on offspring F:B. The model response F:B is log_n_-transformed to meet model requirements and the predictors *Early-PreGC, Late-PreGC, PostGC,* and *OffspringGC* are log_n_-transformed and then z-transformed with mean = 0 and SD = 1 to meet model requirements and to increase model interpretability. Each panel is plotted with all other model predictors at their average value

We detected seasonal differences in the effect of maternal and offspring GCs on the *Firmicutes* to *Bacteroidota* ratio (*Firmicutes*/*Bacteroidota*). While the full model on the *Firmicutes*/*Bacteroidota* during the lean season revealed no significant effect of all model predictors (full-null model comparison: full-Model Lean: *χ*2 = 11.95, *df* = 10, *p* = 0.335, Supplementary Table 5), the models on the *Firmicutes*/*Bacteroidota* during the rich season revealed an overall effect of all predictors together (full-null model comparison: full-Model Rich: *χ*2 = 34.01, *df* = 10, *p* < 0.001, Supplementary Table 5). We excluded the non-significant interaction Late-preGC*Age (*p* = 0.131) and ran a reduced model including the main term *Late-preGC*. Again, the reduced model revealed an overall effect of all model predictors on the *Firmicutes*/*Bacteroidota* ratio during the rich season (reduced-null model comparison: reduced-Model Rich: *χ*2 = 31.73, *df* = 9, *p* < 0.001, Table 4). In particular, both the *Early-preGC* moderated by Age (Early-preGC*Age) and *Late-preGC* as a main term were negatively associated with *Firmicutes*/*Bacteroidota* (Fig. 6A and C; Table 4), while the predictors associated with postnatal maternal GCs and offspring GCs had a positive effect (Fig. 6B and C, Table 4).

**Table 4:** Effect of model predictors on *Firmicutes* to *Bacteroidota* ratio

**Predictor**	**red-Model Rich (pseudo-*R***^**2**^** = 0.86)**				
	**Estimate**	**SE**	**95% C.I**	**LRT**	***p*-value**
Intercept	1.165	0.007	1.152 to 1.178	-	-
Early-preGC^a^	−0.020	0.008	−0.036 to −0.005	-	-
Late-preGC^a^	−0.015	0.006	−0.026 to −0.004	5.624	**0.018**
PostGC^a^	0.011	0.008	−0.005 to 0.027	-	-
OffspringGC^a^	0.033	0.008	0.018 to 0.048	-	-
Age^b^	−0.049	0.011	−0.070 to −0.028	-	-
Sex[M]^c^	0.004	0.009	−0.013 to 0.021	0.177	0.674
^a^Early-preGC*Age^c^	−0.027	0.008	−0.042 to −0.012	9.066	**0.003**
^a^PostGC*Age^c^	0.035	0.008	0.019 to 0.051	11.955	**0.001**
^a^OffspringGC*Age^c^	0.016	0.008	0.001 to 0.031	3.976	**0.046**

Reduced model explaining the *Firmicutes* to *Bacteroidota* ratio of offspring’s gut bacteria during the rich season (red-Model Rich)

^a^Indicates that the predictor has been log_n_-transformed and then z-transformed (mean = 0, SD = 1) to meet model requirements and to increase model interpretability

^b^The predictor has been z-transformed with mean = 0 and SD = 1

^c^Coded with “female” as reference category. *N* = 16

### Maternal GCs and offspring gut bacterial functional profile

The taxonomic changes associated with prenatal maternal GC during early gestation and age (Early-preGC*Age) corresponded to changes in the predicted function of Assamese macaque’s gut microbiota, as predicted by PICRUSt2. The predicted functions of the gut bacterial community during the lean season were only very weakly associated with variation in our predictors (Table 5).

**Table 5:** Effect of maternal GC on predicted microbial functional pathways (KOs at level 3) controlling for the effect of *OffspringGC* and *Sex*. Grey color indicates limited interpretability of individual predictors due to their computed interaction effect

**Predictor**	**Rich Season**		**Lean Season**	
	**Significant KOs**	**Significant KOs (%)**	**Significant KOs**	**Significant KOs (%)**
Early-PreGC	49	15	7	2
Age	28	8	67	20
Late-PreGC	52	16	14	4
PostGC	20	6	12	4
OffspringGC	81	24	0	0
Sex[M]	8	2	0	0
Early-PreGC*Age	64	19	9	3
Late-PreGC*Age	30	9	0	0
PostGC*Age	103	31	4	1
OffspringGC*Age	28	8	0	0

**Fig. 7:**
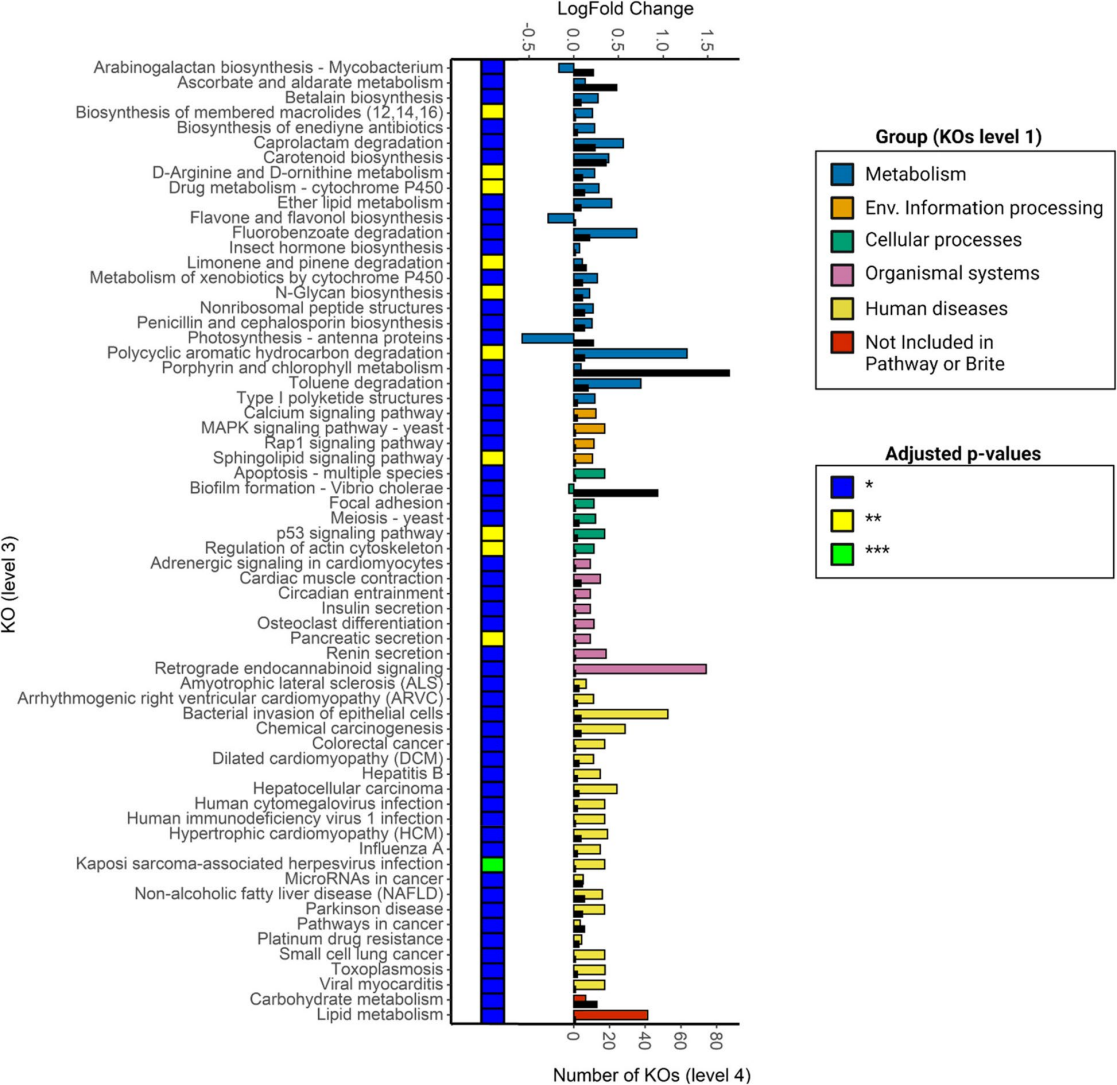
Predicted functional pathways significantly affected by the prenatal maternal GCs and offspring age (Early-PreGC*Age). Colored bars indicate the log-fold change associated with an increase of 1 unit of EarlyPreGC*Age. Black bars indicate the number of KOs grouped at level 4 which contribute to each pathway. Heatmap indicates the significance of each pathway (BH adjusted *p*-values)

During the rich season, Early-preGC*Age was associated with the enrichment of metabolic pathways and pathways involved in different types of diseases. Mostly enriched pathways involved terpenoid and polyketide metabolism, signal transduction processes by which cells detect and respond to changes in their environment, and lipid and carbohydrate metabolism (Fig. 7).

Late-preGC*Age was mostly associated with changes in glycan biosynthesis, characterized by a decrease in predicted Lipoarabinomannan and Arabinogalactan biosynthesis pathways and an enrichment of N-Glycan biosynthesis pathways. Late-preGC*Age was also associated with the enrichment of several terpenoid and polyketide metabolic pathways and a reduction in secondary metabolites. In addition, Late-preGC*Age was associated with a weak enrichment of the predicted pathways involved in amino acid metabolism (Supplementary Fig. 1). Maternal GCs during the first six months of lactation and offspring age (PostGC*Age) was associated with a general enrichment of the predicted pathways involved in bacterial infectious diseases (e.g., Shigellosis, *Escherichia coli*, and *Staphylococcus aureus* infections), alteration of secondary metabolites biosynthesis, and reduction of predicted terpenoid and polyketide metabolism. We observed also alteration of predicted microbial pathways involved in signal transduction, pathways of glycan biosynthesis and metabolism, and amino acid decreased, while we observed enriched lipid metabolic pathways (Supplementary Fig. 2). Offspring GC and age (OffspringGC*Age) was associated with a reduction in predicted microbial pathways associated with lipid metabolism (lower ether lipid metabolism and fatty acid degradation), metabolism of cofactors and vitamins, and with predicted infection of *Staphylococcus aureus* which decreased with age (Supplementary Fig. 3).

Finally, the overall effect of *Early-preGC* amplified with age in almost all the predicted functional microbial pathways contrarily to the effect of *Late-preGC* and *PostGC* which reversed with age, while the effect of *OffspringGC* was equally amplified and reversed with age (Table 6). The interpretations on the effects of variation in putative functional profiles associated with *Late-preGC, PostGC*, and *OffspringGC* must be taken carefully due to their strong variation mediated by age.

**Table 6:** Effect of maternal and offspring GCs on predicted gut bacterial functional pathways

**Predictor**	**KOs significantly affected**	**Amplify with age (%)**	**Attenuate with age (%)**	**Reverse with age (%)**
Early-preGC	64	96.9	0.0	3.1
Late-preGC	30	16.7	6.6	76.7
PostGC	103	5.8	7.8	86.4
OffspringGC	28	42.9	17.8	39.3

Effect of maternal and offspring GCs moderated by age on the predicted gut bacterial function during the rich season (KOs grouped at level 3). The direction of moderation (amplification, attenuation, reversal) is summarized for significantly affected KOs only

## Discussion

Cercopithecoid primates and humans share many similarities in their gut microbiomes, even more than apes and humans [62–64], making macaques a valuable model species for gut microbiome studies. For example, the microbial butyrate production pathways of catarrhines, including macaques, are more similar to those of humans than those observed in lemurs, platyrrhines, and apes after controlling for the effects of dietary strategy [62]. The high microbial diversity associated with the butyrate pathways of catarrhines and humans implies a more efficient short-chain fatty acid (SCFA) metabolism and may confer greater stability and resilience to perturbations. A higher diversity of microorganisms involved in amino acid degradation may confer stability in the face of large variations in diet composition, as seen in comparative butyrate-degradation patterns of industrialized and non-industrialized human populations [62]. The ecological and dietary flexibility of macaques is unique among non-human primates and is thought to underlie the widest latitudinal and longitudinal geographic distribution of all primate genera except *Homo* [65–68]. In addition, unlike germ-free mice, Assamese macaques offer a unique advantage for studying microbiota development in offspring in their natural context by providing a more accurate representation of microbiota development in a natural setting.

In the following, we first discuss the broad effects of age and season on the gut bacterial composition and then focus on the timing effects of maternal GC exposure and on the outcome in terms of known functions of taxa most profoundly affected by maternal GCs in our statistical models.

### Seasonal and age differences on gut microbiome composition

Assamese macaques at the Phu Khieo Wildlife Sanctuary (Thailand) can be classified as frugivorous, as they consume mainly fruit, pulp, and seeds, with no marked age- or sex-related differences in the diet composition of plant parts [42, 43]. However, they spend a considerable part of their feeding time opportunistically and slowly foraging for animal matter, regularly consuming large quantities of aquatic mollusks, spiders, ants, caterpillars, termites, and less regularly small snakes and lizards, amphibians, birds and bird eggs, and even small mammals including several species of rats and squirrels [43]. Assamese macaques from Phu Khieo Wildlife Sanctuary consume at least 165 known plant items belonging to 118 plant species with a moderate overlap of the most common items between years [42]. As environmental conditions in forests of south-east Asia are highly unpredictable, the predictability of food and fruit abundances are also very low. Our study species, therefore, evolved to live in a highly unpredictable environment [44] and although the main part of its diet is based on plant parts, their gut microbiome must endure unpredictable variation in food type ingested which inevitably has to rely on and may benefit from higher gut microbiota diversity.

Among primates, age-related patterns of microbial richness variation are diverse, and the wild Assamese macaques investigated in this study appear to be more similar to humans than other genetically closer species like chimpanzees [69]. Our results showed an increase in bacterial richness from infants via juveniles to adults and are in line with studies on vervet monkeys, rhesus macaques, and humans [70–72] but contrast with observations in wild chimpanzees, common marmoset, and mouse lemurs [69, 73, 74].

Across age and seasons, the sum of the relative abundances of *Lachnospiraceae, Prevotellaceae, Spirochetaceae, Ruminococcaceae,* and *Oscillospiraceae* was approximately 50%, making these families the most abundant ones in wild Assamese macaques. These groups comprise cellulolytic and fermentative taxa that can metabolize complex plant polysaccharides and degrade fibers. The higher abundance of *Prevotella* is a common trait of folivore/frugivore species and produces twice more propionate than bacterial communities dominated by *Bacteroides* [75]. As distinctive traits of most macaque species, also wild Assamese macaques revealed a high abundance of *Spirochetaceae* from the *Treponema* lineage, a genus whose functionality in the gut is still unexplored. Yet, many *Treponema* are fiber-degrading bacteria proposed to give a substantial contribution to short-chain fatty acid (SCFA) production together with *Prevotella* by degrading xylan, xylose, and carboxymethylcellulose [76]. It is absent in humans living in urban environments but inhabits the gut of individuals from rural communities consuming termites [77].

**Fig. 8:**
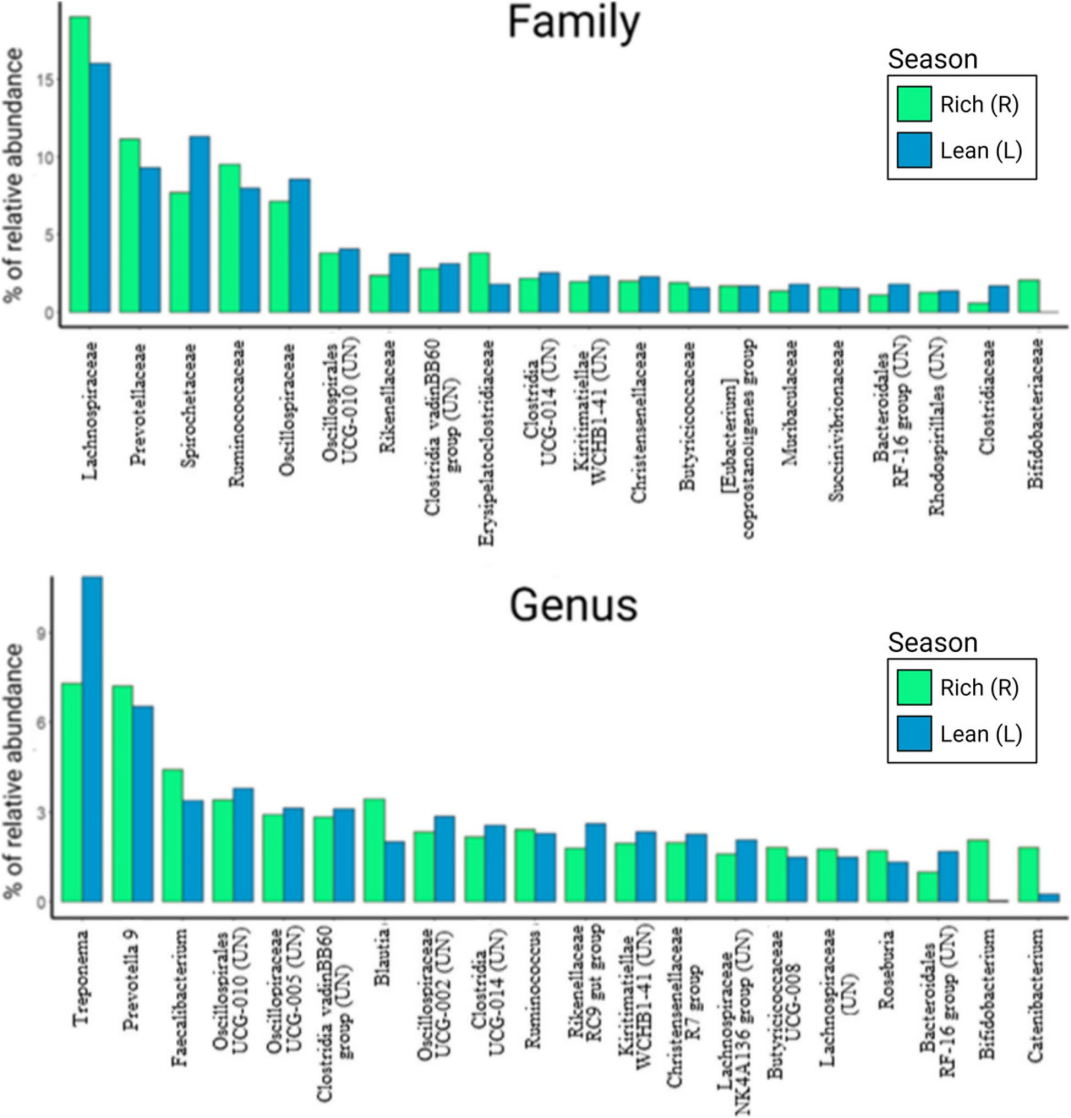
Relative abundance of the 20 most abundant (top) families and (bottom) genera observed in fecal samples of wild Assamese macaques collected during the rich (R, green bars) and the lean season (L, blue bars) at the Phu Khieo Wildlife Sanctuary (Thailand). All the panels have been plotted with R and edited in Biorender.com

Across seasons, the relative abundance of the top 20 taxa at the family or genus level did not differ markedly except for some taxa (Fig. 8): the rich season showed a higher relative abundance of the genera *Blautia* and *Roseburia (Lachnospiraceae)*, *Faecalibacterium*, *Catenibacterium,* and *Bifidobacterium,* and the families *Prevotellaceae* and *Bifidobacteriaceae,* while the lean season showed higher relative abundance of *Spirochetaceae*, *Rikenellaceae*, *Oscillospiraceae,* and *Clostridiaceae* (Fig. 8).

Typically, members of the *Cercopithecidae* show low relative abundance and diversity of *Bifidobacterium* compared to other primates [78, 79]. Yet, despite host-phylogenetic differences, *Bifidobacterium* is strongly affected by diet [79]. It is highly abundant in the gut of breastfed humans and nursing non-human primate infants and has a key role in the digestion of complex carbohydrates from milk [69, 80]. In the rich season of this study, when infants were still highly dependent on milk, *Bifidobacterium* relative abundance in wild Assamese macaques was highest in infants and decreased with age, it had a relative abundance of 3% across all age classes, but was almost absent in samples from the lean season. Higher abundance during the rich season may be driven by hosts consuming fruits and enriching their diet with animal matter like Assamese macaques having a higher abundance of *Bifidobacteriaceae* when compared with folivores, frugivores, and omnivores [78, 79]. Future investigations should focus more on direct quantification of animal matter consumption during the rich and the lean season to clarify seasonal variation of *Bifidobacterium’s* relative abundance.

We observed seasonal variation also in the abundance of *Catenibacterium* which was higher during the rich season. Organisms belonging to this taxon can harvest a high quantity of energy through the fermentation of carbohydrates and produce several SCFAs like butyrate, lactate, and acetate [81]. *Catenibacterium* has a higher relative abundance in wild long-tailed macaques when compared with captive individuals [82] and its increase is associated with higher energy storage in humans and rodent models. Thus, members of this genus can have a relevant role in the energy accumulation strategy of wild Assamese macaques which must be maximized during the rich season, the best period of the year for energy storage.

### The timing effect of pre- and postnatal maternal GCs on gut bacterial composition and function

The effect of maternal stress on the programming of physiological phenotypes is a critical factor strongly influenced by the timing of exposure [1, 36, 44, 83–87]. We tested for the first time in a wild and long-lived species the effect of maternal GCs on bacterial diversity, composition, and predicted functional pathways according to three sensitive time windows: early gestation, late gestation, and postnatal maternal GCs during lactation. The effect of maternal GCs on gut bacterial alpha diversity varied according to the timing of exposure. Analyses consistently revealed a negative effect of prenatal maternal GCs during early gestation (*Early-preGC*) on gut bacterial diversity, but no significant effects from maternal GCs exposure during late gestation (*Late-preGC*) or postnatally during lactation (*PostGC*). Our results on the effect of naturally driven maternal GCs in wild individuals are also in line with studies on primates and rodents in captivity reporting a reduction in infants’ microbial diversity associated with experimentally induced stress during gestation [4, 36, 39]. Fetal exposure to maternal GCs affects HPA axis development and changes gut permeability by perturbing barrier functionality with consequent alteration of typical microbial colonization of the gut [2, 4, 5, 10, 17, 88–92].

The composition of gut bacteria during the lean season was only very weakly associated with variation in our predictors. During the rich season, however, bacterial composition was considerably changed with changing levels of maternal GCs and offspring age with a quarter of taxa being differentially abundant. While the proportion of taxa significantly affected was roughly similar for all pre- and postnatal phases, the effects either faded with age or reversed with age in response to *Late-preGC* or *PostGC*. In response to *Early-preGC*, the effects intensified with age (positively or negatively) in the vast majority of differentially abundant taxa.

Similarly, the PICRUSt2 predicted functional profiles of the gut bacteria during the lean season were very weakly associated with variation in our predictors. Instead, in samples collected during the rich season, we observed a significant variation in nearly 20% of the predicted functional pathways associated with variation in *Early-PreGC* which intensified with age. Again, the overall effect of maternal GC during late gestation was weaker than the effect during early gestation and tended to reverse with age. Although we observed a considerable overall effect of maternal GC during lactation on predicted microbial pathways this effect strongly reversed with age.

Thus, the timing of GC exposure had a pronounced effect on the persistence of a long-term gut bacterial signature. Our results on the timing effect of prenatal maternal GCs are in line with other studies that highlight the important role of maternal GC during early as opposed to late gestation in programming morphological or physiological phenotypes in long-lived primates [1, 33, 44] and expand the list of potentially detrimental effects programmed during the very first phases of fetal development.

### Long-term signature of early prenatal maternal GCs

Early preGCs were not only associated with reduced gut bacterial richness all the way into adulthood but also with a previously unstudied reduction or increase in the relative abundance of 14% of the total genera that amplified with age. We observed a reduction in the abundance of *Bifidobacterium, Acetivibrio, Succinivibrio, Olsenella,* and *Slackia,* and this reduction intensified with age. Typically, *Bifidobacterium* abundance decreases with age in humans and non-human primates [93–95]. The presence of this health-promoting organism in the gut inhibits pathogen attachment to intestinal cells, promotes protection against enterocolitis and acute diarrhea by slowing down the replication of enteric pathogens, and is typically involved in folate biosynthesis and antioxidant production in human and non-human primates [72, 78, 79]. Long-term physiological stimulation of the innate immune system can be detrimental with age and can lead to inflammaging processes [96]. Our results suggest that prenatal maternal GCs effects on gut bacteria mimics the inflammaging process which would corroborate findings in rhesus macaques showing a signature of inflammaging in differential gene expression after exposure to the ecological effects of a recent hurricane [97]. The gut microbiome has a pivotal role in regulating these processes and an increase in the abundance of *Bifidobacterium* has been associated with improved health and longevity and with a reduction or even reversal of dysfunctions driven by inflammatory states associated with age [96]. During the rich season, older subjects were affected more negatively by early prenatal maternal GCs than younger ones. Thus, prenatal maternal GCs may accelerate the typical reduction in *Bifidobacterium* associated with age [93] in Assamese macaques with consequent long-term dysbiotic states in adult subjects which may benefit less from the health-promoted effect of this taxon. The wider age window for offspring in this study from infants to adults may explain discrepancies between these findings and a study on captive rhesus macaques confined to only infant offspring where not the early but the late prenatal maternal exposure to stress was associated with decreased relative abundance of *Bifidobacterium* [39]. Among the putative pathways of bacterial infectious diseases, we observed an increase in signals of bacterial invasions of epithelial cells, the adhesins/invasins. Both involved in the process of adhesion and invasion of host cells and are important virulence factors that enable microorganisms to cause disease. We believe this to be an indication that prenatal maternal GCs may accelerate the increase of susceptibility to infectious diseases normally associated with age. However, since we did not observe a significant increase in pathogenic bacteria associated with *Early-PreGC*, these results on gut bacterial function and infectious diseases need to be interpreted carefully.

Furthermore, we observed a reduction in the genus *Oscillospira* and an unclassified member of *Oscillospiraceae* with increasing early prenatal maternal GCs and age. *Oscillospira* is one of the most abundant genera observed in many primate species [98–103], including humans, and it has recently been proposed as a potential probiotic [104]. It is a slow-growing butyrate producer that ferments complex plant carbohydrates, can utilize host glycans as an energy source, and its abundance is negatively associated with pro-inflammatory molecules in rodent models [105] and with inflammatory disorders in humans [106–108], it is reduced in non-human primates with infections, in captivity or in individuals with higher inflammatory responses to cerebral infarction [14, 24, 102]. *Oscillospira* increases with age in several macaque species [100, 101, 109] and it is positively associated with bacterial diversity in humans [107]. Interestingly, wild Assamese macaques with higher prenatal GCs exposure showed lower *Oscillospira* abundance and richness, the same pattern observed in non-healthy humans [110, 111], and in captive or inflamed non-human primates [14, 24, 102], and lacked the typical increase observed with age in healthy individuals of other macaque species [100, 101, 109]. These results suggest a role of maternal GCs in altering the typical colonization of the abundant *Oscillospira,* with potential long-term detrimental effects in subjects with higher prenatal GCs exposure.

Additionally, the bacterial signature of increased early prenatal maternal GCs was characterized by an increase in the relative abundance of several pro-inflammatory organisms that amplified with age. We observed an increased abundance of a member of the *Enterobacteriaceae* and *Enterococcaceae*, aerotolerant taxa whose overgrowth is promoted by inflammation [112], *Tyzzerella*, a typical pro-inflammatory bacterium which increases intestinal permeability and correlates with circulating inflammatory outcomes and anxiety-like behavior [113–115]. Furthermore, early prenatal GC exposure was associated with an increase in the abundance of the pathobiont *Gemella,* a genus with Gram-positive facultative anaerobic organisms involved in gut inflammation [116], associated with an increased risk of all types of diabetes due to the production of endotoxins inducing inflammatory states [117, 118], and able to cause several diseases (e.g., endocarditis, sepsis, allergies, and asthma) [119].

Notably, we observed differences in two putative endocrine system functions which may be potentially altered by the gut microbiota—Early-PreGC*Age significantly increased the insulin and renin secretion pathways. Although we did not observe evidence of a direct effect on insulin resistance (adjusted *p*-value = 0.921), the sphingolipid signaling pathway was enriched. Among mammals, sphingolipids are fundamental structural and bioactive signaling molecules with important roles in the development of metabolic disorders and gut microbiota derived sphingolipids have been shown to signal into inflammation-related pathways in the colon [120–124]. Thus, it is possible that prenatal maternal GC exposure may translate into an epigenetically driven increase in gut-bacteria sphingolipid signaling pathways that could be associated with later chronic inflammatory states. In general, these predictions should be interpreted cautiously until our preliminary results are confirmed by direct testing. The long-term signature of early prenatal maternal GCs mediated by age seems to be associated with a plethora of pathways and signals indicative of increasing inflammatory states, disease, and cancer. Although this scenario is plausible, these results need to be considered with caution until direct tests on host inflammatory states and health will be conducted to validate the scientific merit.

Interestingly, a genus that changed against the pattern of decreased anti-inflammatory and increased pro-inflammatory taxon abundance was *Butyricimonas*—a butyrate producer that increased in relative abundance with *Early-preGC* and *Age*. Butyrate reduces inflammation in the gut, helps maintain gut integrity [125], and protects against infections [126]. Butyrate producers therefore contribute to the gut homeostasis by increasing the production of mucin which improves tight junction integrity [127]. Such findings could indicate a potential activation of the host immune function in Assamese macaques with higher prenatal GCs which may suffer from a reduced microbial richness and potential associated metabolic pathways and may rely mainly on fewer bacterial species to digest plant and animal matter, to harvest and store energy, and to produce SCFAs. Analyses of predicted microbial function showed no evidence of a significant decrease in the butyrate production and amino acid production in offspring with increasing *Early-preGC.* Thus, one interpretation is that a higher relative abundance of a few bacterial species that produce SCFAs and amino acids may compensate for the lack of diversity in SCFAs and amino acid producers. However, according to life-history theory, resource diversification may be adaptive or maladaptive depending on the environmental context [128]. In an unpredictable environment, like the ones Assamese macaques live in, putting all eggs in one basket represents a high risk, which can only be clarified by studies on the mediation role of the gut microbiome on the effect of prenatal maternal stress on fitness. Despite this one outlier, patterns of differential abundance associated with prenatal GC exposure are suggestive of inflammatory states exacerbating with age.

Other marked changes that amplified with offspring age when *Early-preGCs* increased were decreased abundances of taxa with metabolic functions like energy harvesting and storage. *Acetivibrio* is known to produce SCFA from fibers [129], and *Succinivibrio* is a glucose-fermenting organism promoting cellulose and hemicellulose digestion, producing succinic and acetic acids and helping in fatty acids metabolization which can increase the efficiency in energy utilization [130, 131]. We also observed a significant reduction in *Olsenella, Collinsella,* and an uncultured member of the *Erysipelotrichaceae*, all taxa associated with energy storage in a high-fat diet [63, 132–136] and with increased fruit consumption in gorillas and humans [137].

The gut bacteria of early prenatally challenged offspring was further characterized by a significant decrease in *Firmicutes*/*Bacteroidota* ratio. Their relationship is associated with lipid metabolism and an increase in their ratio is indicative of a more efficient capability of energy harvesting and a greater provision of beneficial SCFAs [82, 138]. A recent study investigating oral and gut bacteria, and body fat indices in wild versus captive long-tailed macaques revealed that wild subjects harbored higher gut and oral diversity, higher *Firmicutes*/*Bacteroidota* ratio, and had higher body fat indices when compared with captive individuals [82]. It is possible that individuals with higher prenatal maternal GCs may have an overall reduced capability of energy harvesting similarly observed in captive and leaner long-tailed macaques.

Being relaxed income breeders, Assamese macaques accumulate fat during the pre-mating season and rely on the rich season to store the fat necessary to energetically support the next gestation or the current lactation [41]. Early lactating females and those that are neither gestating nor lactating follow an energy-conserving strategy by storing as much fat as possible during the rich season [41] to ultimately increase their reproductive success. Indeed, fruit availability at Phu Khieo Wildlife Sanctuary modulates conception rates, the peak of the energetically costly lactation period overlaps with the peak of fruit availability, and females exhibit poor physical condition during lactation [42]. Typically, females in our study population need time to recover from previous reproductive effort and skip reproduction in a given year if they gave birth late in the previous birth season [42]. As the best period to store energy is during the rich season, prenatal maternal stress can negatively impact the energy storage strategy during the fundamental step grounding Assamese macaques’ reproductive success by decreasing the relative abundance of several bacterial taxa involved in energy storage and SCFAs production. Although we observed significant alterations of putative metabolic pathways, our results on microbial function did not support these hypotheses. Most of the well-known microbial pathways involved in energy harvesting and storage were not affected by early prenatal maternal GC (e.g., starch and sucrose metabolism, metabolism of pyruvate and propanoate, pentose phosphate pathway, glycolysis/gluconeogenesis, fatty acid biosynthesis, glycerophospholipid metabolism, linoleic acid metabolism). However, we observed variation in carbohydrate and lipid metabolic pathways that are not yet fully categorized in the KEGG database. Among the putative genes involved in the unclassified carbohydrate metabolism category, there were KOs related to the D-lactate dehydrogenase, myo-inositol catabolism, and acetoin utilization protein. Lactate dehydrogenase enzymes play an important role in cellular metabolism, helping to convert lactate to pyruvate and supporting the production of energy for the bacteria in the form of ATP. Myo-inositol catabolism is an important metabolic pathway for bacterial growth and survival allowing bacteria to utilize a wide range of carbon sources to generate ATP. Instead, acetoin is secreted during the exponential growth phase and also serves as a carbon storage compound which can be reused when other carbon sources have been exhausted. The ability of gut bacteria to utilize acetoin as an energy source is important for their survival in the gut environment, where there is competition for nutrients among different microbial species. Notably, acetoin it is also considered a “metabolic toxin” that can chemically modify host signaling molecules, such as hormones and neuro-transmitters, thereby affecting their biological activity and it has been suggested that increased production of acetoin by gut bacteria is associated with conditions such as inflammatory bowel disease [139, 140]. Further investigations on such uncategorized metabolic pathways and analysis of fecal SCFA profiles in Assamese macaques will help clarify how prenatal maternal GCs during early gestation alter gut microbiota metabolism of carbohydrates and lipids and its implications for host health.

**Fig. 9:**
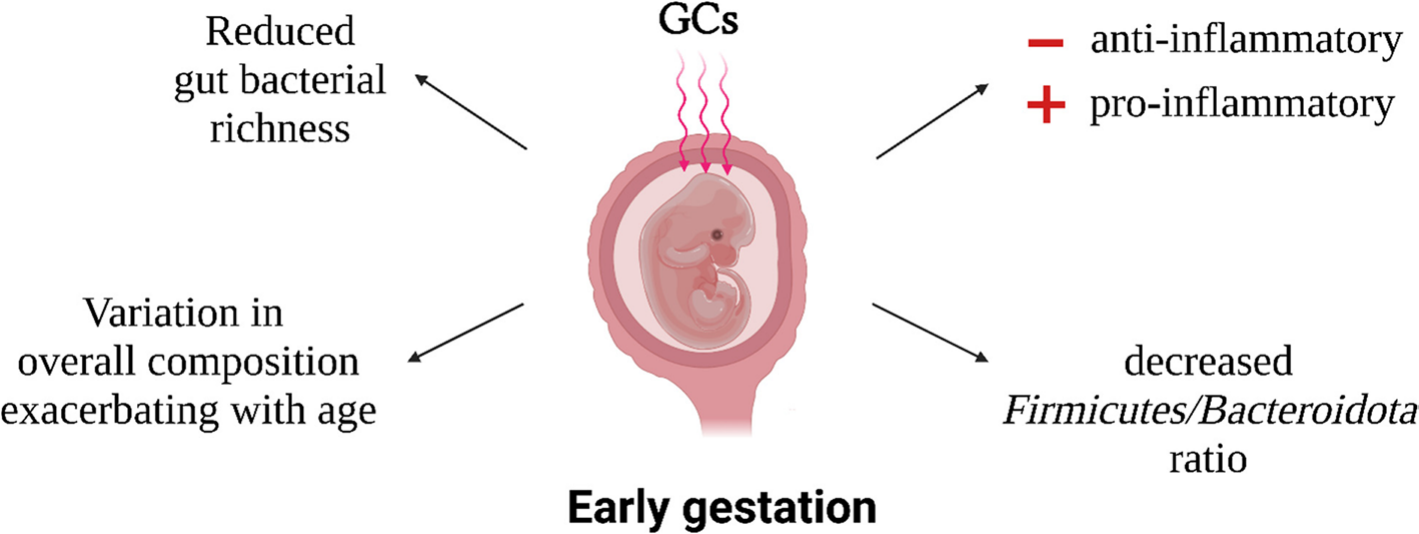
Microbial signature of maternal GCs during early-gestation observed across infant, juvenile, and adult age in wild Assamese macaques during the rich season. Exposure to maternal GC late during gestation or after birth had much weaker or less stable effects. Made with Biorender.com

In summary, the long-term signature observed in the gut bacterial community and associated with the exposure to maternal GC early during gestation (Fig. 9) affects the balance of pro-and anti-inflammatory taxa.

It seems unlikely that in this study, the effects of early maternal GC exposure on gut bacteria were mainly mediated by increased HPA axis activity in challenged offspring. Both the early developmental and the concurrent offspring GC levels were included in all the statistical models. Variation inflation factor analyses suggest that their possible covariation in this dataset does not affect the significance of *Early-preGC* as a predictor. Offspring GC did not explain residual variation in gut bacterial community diversity and its effects on community composition were less broad on the phylum and family level and less stable across offspring age at the genus level. Thus, we believe that early prenatal maternal effects on gut bacteria are more likely to be mediated by alterations of gut microanatomy, physiology, and immunology of the epithelial wall including changes in permeability.

### Offspring GCs and gut bacteria

Measures of offspring GCs were not associated with gut bacterial species richness measured from the same fecal sample in wild Assamese macaques which corroborates results on wild gorillas [11, 63] and Verreaux’s sifakas [141], but contrasts with other studies showing a negative effect in red squirrels [142, 143]. Quantitatively, offspring GC had an age-moderated effect on genus composition during the rich season only slightly smaller than that of prenatal and postnatal maternal GCs. However, the effect was less stable and less pronounced than *Early-preGC* and was similar to what observed in the function analyses results. Focusing on the effects that amplified with age, increasing seasonal offspring GC levels were associated with a higher relative abundance of the genus *Victivallis* and a lower abundance of unclassified members of *Clostridia UCG-014* and *Lactobacillaceae*, *Anaerosporobacter*, *Streptococcus, Paludicola,* and *Ruminiclostridium.* Unfortunately, the information on metabolic function and potential pathogenicity of most of these organisms is still unexplored. *Ruminiclostridium* can secrete SCFAs; in the herbivore black goat, it is considered a potential beneficial bacterium positively involved in growth regulation and gut permeability and helps in maintaining homeostasis and morphology of intestine epithelial cells [144]. *Streptococcus* is a well-known genus with not only several potential pathogens but also beneficial species. Together with *Bifidobacterium,* members of the *Streptococcus* genus are commensal and are the major producers of lactic acid and acetate in humans with protection against enteropathogenic infection and inflammation reduction [145, 146]. *Streptococcus* is associated with increased degradation of fibers and increased production of SCFAs [147]. In obese humans, a reduction in beneficial *Streptococcus* was observed in subjects with a higher intake of dietary fructose [148]. A lower presence of *Streptococcus* could be associated with a lower production of beneficial SCFA and fiber degraders or a lower abundance of potential pathogens. However, our preliminary analyses of the age-mediated effects of offspring GC on putative gut bacterial functions also showed no evidence of strong variation in the metabolic functions. Notably, we observed a positive effect on potential *Staphylococcus aureus* infection pathway which, however, faded out with age, suggesting a potential detrimental effect interesting mostly infant and juvenile macaques. Finally, higher offspring GC levels were associated with higher *Tyzzerella* in infants and juveniles but not in adults suggesting an increase of a potentially pathogenic organism in young subjects with higher GCs which fades with age. However, pathogenicity confirmation requires genomic data with higher resolution at the strain level and was not part of this study.

GCs are metabolic hormones providing information on energy mobilization in response to energetic challenges. The stress response characterized by the release of GCs is an adaptive response to maintain physiological homeostasis during adverse conditions [149] including energy deficits [41]. During the rich season, higher offspring GCs levels were associated with higher *Firmicutes*/*Bacteroidota* ratio suggesting higher energy harvesting capability [150]. As the rich season represents the best time for wild Assamese macaques to store energy, an energy deficit is possible but unlikely. In the same study population and in the same individuals, prenatal maternal GCs induced a recalibration of offspring HPA axis activity with long-term hyperactivation and higher allostatic load in infants, juveniles, and adults [30]. One possibility is that if prenatally stressed individuals maintain a persistent hyperactivity of the HPA axis, perhaps a gut microbiota with enhanced capability of energy harvesting may serve to offset some of these costs. Rather puzzlingly, PICRUSt2 analyses of putative gut bacterial functions showed no evidence of potential effects of offspring GCs on energy harvesting or storage, leaving us with more questions than answers about host stress-induced changes in gut microbiome metabolism. Today, there is a need to better understand how variation in gut bacterial composition may affect energy balance, and future studies could focus on the investigation of metabolic markers to disentangle the role of the gut microbiota in mediating potential energetic constraints and adaptive strategies associated with early adversity.

## Conclusion

In this study, we added ecological validity to models about prenatal stress and gut microbiome alterations tested in captivity and under controlled environmental conditions. Using multivariate regression models and ANCOM-BC approach on a cross-sectional sample, we assessed for the first time in a wild long-lived animal species the effect of maternal GCs on gut bacterial diversity, function, and composition of infant, juvenile, and adult offspring. Timing of the exposure was critically associated with variation in prenatal effects, and early gestation was confirmed as a fundamentally sensitive period for shaping offspring physiological phenotypes with persisting long-term effects. From infancy to adulthood, we observed a bacterial signature and dysbiotic states of prenatal maternal GCs characterized by an overall reduction in bacterial richness, an imbalance in the ratio of the detrimental to beneficial organisms with an increase of potentially pro-inflammatory organisms. The effect of maternal GCs during early gestation was more evident during the rich season, a pre-mating time window important for energy accumulation in Assamese macaques and necessary to support energetically the current lactation or the upcoming reproduction. Interestingly, higher offspring GC levels had no negative effect on richness and, with the caveat of being a correlational study, the prenatal maternal effects seem to be rather independent instead of being mediated by offspring GC levels. Finally, the bacterial signature under the effect of prenatal maternal GCs was characterized by a potential reduction in resource diversification with a higher potential risk for detrimental health and reduced fitness in an unpredictable environment.

## Supplementary Information

**Additional file 1.** Amplification of 16S rRNA genes and sequencing: full procedure. **Supplementary Table 1.** Full models 1a-1c explaining the offspring’s gut bacteria alpha diversity (from richness estimators). Significant *P* values of explanatory variables are in bold. *R*^2^ indicates the conditional coefficient of determination. All covariates are log_n_-transformed and then z-transformed (mean = 0, SD = 1) to meet model requirements and increase model interpretability. ^(1)^ coded with “female” as the reference category, ^(2)^ coded with “infant” as the reference category, ^(3)^ coded with “lean” as the reference category, ^(4)^ coded with the group “MOT” as the reference category. **Supplementary Table 2.** Full model 1d-1e explaining the offspring’s gut bacteria alpha diversity (from richness-evenness estimators). Significant *P* values of explanatory variables are in bold. *R*^2^ indicates the conditional coefficient of determination. All covariates are log_n_-transformed and then z-transformed (mean = 0, SD = 1) to meet model requirements and increase model interpretability. ^(1)^ coded with “female” as the reference category, ^(2)^ coded with “infant” as the reference category, ^(3)^ coded with “lean” as the reference category, ^(4)^ coded with the group “MOT” as the reference category. **Supplementary Table 3.** Reduced models 1a-1c explaining the offspring’s gut bacteria alpha diversity (from richness estimators). Significant *P* values of explanatory variables are in bold. The model was tested via model comparison with null models: Model 1a: χ2 = 72.85, df = 11, *p* < 0.001; Model 1b: χ2 = 91.98, df = 11, *p* < 0.001; Model 1c: χ2 = 66.38, df = 11, *p* < 0.001. *R*^2^ indicates conditional coefficient of determination. All covariates are log_n_-transformed and then z-transformed (mean = 0, SD = 1) to meet model requirements and increase model interpretability. ^(1)^ coded with “female” as the reference category, ^(2)^ coded with “infant” as the reference category, ^(3)^ coded with “lean” as the reference category, ^(4)^ coded with the group “MOT” as the reference category. **Supplementary Table 4.** Reduced model 1d and 1e explaining the offspring’s gut bacteria alpha diversity (from richness-evenness estimators). Significant *P* values of explanatory variables are in bold. The model was tested via model comparison with null models: Model 1d: χ2 = 29.26, df = 15, *p* = 0.015; Model 1e: χ2 = 20.08, df = 11, *p* = 0.044. *R*^2^ indicates the conditional coefficient of determination. All covariates represent average values log-transformed and then z-transformed. All covariates are log_n_-transformed and then z-transformed (mean = 0, SD = 1) to meet model requirements and increase model interpretability. ^(1)^ coded with “female” as the reference category, ^(2)^ coded with “infant” as the reference category, ^(3)^ coded with “lean” as the reference category, ^(4)^ coded with the group “MOT” as the reference category. **Supplementary Table 5.** Full linear models explaining the *Firmicutes* to *Bacteroidota *ratio of offspring’s gut bacteria during the lean (Full-Model L) and the rich season (Full-Model R). ^(^^1)^ indicates that the predictor has been log_n_-transformed and then z-transformed with mean = 0 and SD = 1 to meet model requirements and to increase model interpretability; ^(^^2)^ indicates that the predictor has been z-transformed with mean = 0 and SD = 1; ^(^^3)^ coded with the group “MOT” as the reference category; ^(^^3)^ coded with “female” as the reference category. **Supplementary Table 6.** Effect of *Early-preGC* in interaction with age on gut bacterial composition during the rich season. Infants are excluded from the analyses. UN = unclassified at the genus level. **Supplementary Figure 1.** Predicted functional pathways significantly affected by the prenatal maternal GCs and offspring age (Late-PreGC*Age). KOs are grouped at level 3 of KEGG database. Only significantly affected pathways are plotted (BH adjusted p-values). Colored bars indicate the effect of an increase in 1SD of *Late-preGC* (SD = 67.0 ng/g) estimated at the mean value of age (mean = 4.7 years), and at the mean value of all the other predictors. Red and black lines indicate the effect estimated at the mean plus1SD (red = 7.1 years) and at the mean minus1SD (black = 2.3) of age. When the bar is not plotted, the effect of the same increase in maternal GCs at the mean age is 0. Taxa showing the black head (estimates at 2.3 years) further from 0 than the red head (estimates at 7.1 years) show an effect that is reduced with age. **Supplementary Figure 2.** Predicted functional pathways significantly affected by the postnatal maternal GCs and offspring age (PostGC*Age). KOs are grouped at level 3 of KEGG database. Only significantly affected pathways are plotted (BH adjusted p-values). Colored bars indicate the effect of an increase in 1SD of *PostGC* (SD = 52.7 ng/g) estimated at the mean value of age (mean = 4.7 years), and at the mean value of all the other predictors. Red and black lines indicate the effect estimated at the mean plus1SD (red = 7.1 years) and at the mean minus1SD (black = 2.3) of age. When the bar is not plotted, the effect of the same increase in maternal GCs at the mean age is 0. Taxa showing the black head (estimates at 2.3 years) further from 0 than the red head (estimates at 7.1 years) show an effect that is reduced with age. **Supplementary Figure 3.** Predicted functional pathways significantly affected by the offspring GCs and offspring age (OffspringGC*Age). KOs are grouped at level 3 of KEGG database. Only significantly affected pathways are plotted (BH adjusted p-values). Colored bars indicate the effect of an increase in 1SD of *OffspringGC* (SD = 130.1 ng/g) estimated at the mean value of age (mean = 4.7 years), and at the mean value of all the other predictors. Red and black lines indicate the effect estimated at the mean plus1SD (red = 7.1 years) and at the mean minus1SD (black = 2.3) of age. When the bar is not plotted, the effect of the same increase in maternal GCs at the mean age is 0. Taxa showing the black head (estimates at 2.3 years) further from 0 than the red head (estimates at 7.1 years) show an effect that is reduced with age.

## Data Availability

The datasets generated during and/or analyzed during the current study, and the R codes used to generate analyses and figures, are available in the https://doi.org/10.25625/SFHRIT repository. The raw 16S RNA gene sequencing data are available in the BioProject database (ID: PRJNA795139) of the NCBI repository http://www.ncbi.nlm.nih.gov/bioproject/795139.
