# Supplementary material for: The long-term gut bacterial signature of a wild primate is associated with a timing effect of pre- and postnatal maternal glucocorticoid levels
Source: Microbiome. 2023 Jul 27;11:165. doi: 10.1186/s40168-023-01596-w (PMC10373267; doi:10.1186/s40168-023-01596-w)
Supplement: Supplementary file 2 — Additional file 1. Amplification of 16S rRNA genes and sequencing: full procedure. Supplementary Table 1. Full models 1a-1c explaining the offspring’s gut bacteria alpha diversity (from richness estimators). Significant P values of explanatory variables are in bold. R2 indicates the conditional coefficient of determination. All covariates are logn-transformed and then z-transformed (mean = 0, SD = 1) to meet model requirements and increase model interpretability. (1) coded with “female” as the reference category, (2) coded with “infant” as the reference category, (3) coded with “lean” as the reference category, (4) coded with the group “MOT” as the reference category. Supplementary Table 2. Full model 1d-1e explaining the offspring’s gut bacteria alpha diversity (from richness-evenness estimators). Significant P values of explanatory variables are in bold. R2 indicates the conditional coefficient of determination. All covariates are logn-transformed and then z-transformed (mean = 0, SD = 1) to meet model requirements and increase model interpretability. (1) coded with “female” as the reference category, (2) coded with “infant” as the reference category, (3) coded with “lean” as the reference category, (4) coded with the group “MOT” as the reference category. Supplementary Table 3. Reduced models 1a-1c explaining the offspring’s gut bacteria alpha diversity (from richness estimators). Significant P values of explanatory variables are in bold. The model was tested via model comparison with null models: Model 1a: χ2 = 72.85, df = 11, p < 0.001; Model 1b: χ2 = 91.98, df = 11, p < 0.001; Model 1c: χ2 = 66.38, df = 11, p < 0.001. R2 indicates conditional coefficient of determination. All covariates are logn-transformed and then z-transformed (mean = 0, SD = 1) to meet model requirements and increase model interpretability. (1) coded with “female” as the reference category, (2) coded with “infant” as the reference category, (3) coded with “lean” as the reference category, [file 40168_2023_1596_MOESM1_ESM.docx]

**Supplementary Material of:**

## **The long-term gut bacterial signature of a wild primate is associated with a timing-effect of pre- and postnatal maternal glucocorticoid levels**

Simone Anzà^1,2,3,4^, Dominik Schneider^4^, Rolf Daniel^4^, Michael Heistermann^5^, Somboon Sangmaneedet^6^, Julia Ostner^1,2,3^, Oliver Schülke^1,2,3^

^1^ Behavioral Ecology Dept., University of Goettingen, Goettingen, Germany

^2^ Primate Social Evolution Group, German Primate Center, Leibniz Institute for Primate Research, Goettingen, Germany

^3^ Leibniz ScienceCampus Primate Cognition, German Primate Center, Leibniz Institute for Primate Research, Goettingen, Germany

^4^ Genomic and Applied Microbiology and Göttingen Genomics Laboratory, Institute of Microbiology and Genetics, University of Göttingen, Göttingen, Germany

^5^ Endocrinology Laboratory, German Primate Center, Leibniz Institute for Primate Research, Kellnerweg 4, 37077 Göttingen

^6^ Department of Pathobiology, Faculty of Veterinary Medicine, Khon Kaen University, Khon Kaen, Thailand

**Amplification of 16S rRNA genes and sequencing: full procedure**

Each PCR contained 10 µl of 5-fold Phusion GC buffer, 0.2 µl 50 mM MgCl2, 2.5 µl 5% DMSO, 1 µl 10 mM dNTPs, 31.3 µl nuclease free water (Ambion), 1 µl of forward and 1 µl of reverse primers (equivalent to 0.2 mM), 0.5 µl of Phusion High-Fidelity DNA Polymerase (2 U/µl; ThermoFischer Scientific) and 2.5 µl of 20 ng/µl DNA extract for a total volume of 50 µl. We performed PCR in triplicates on a Labcycler Basic (SensoQuest) with an initial denaturation at 98°C, followed by 25 cycles of denaturation at 98°C for 45 sec, annealing at 55°C for 45 sec and elongation at 72°C for 30 sec. The final elongation was at 72°C for 5 min and samples were then maintained at 10°C until further processing. We confirmed the amplification efficiency and purity by visualizing PCR products on agarose gel electrophoresis. We included negative control without DNA template and a positive control with DNA from *E. coli* on all runs.

After pooling amplicon triplicates, the PCR products were used to attach indices and Illumina sequencing adapters using the Nextera XT Index kit (Illumina, San Diego) and the KAPA HiFi HotStart ReadyMix (Roche Diagnostics, Mannheim, Germany). Index PCR was performed using 5 µl of template PCR product, 2.5 µl of each index primer, 12.5 µl of 2x KAPA HiFi HotStart ReadyMix and 2.5 µl PCR grade water. Thermal cycling scheme was: 95 ° C for 3 min, 8 cycles of 30 s at 95 °C, 30 s at 55 °C and 30 s at 72 °C and a final extension at 72°C for 5 min. Products were quantified using the Quant-iT dsDNA HS assay kit and a Qubit fluorometer (Invitrogen GmbH, Karlsruhe, Germany) following manufacturer’s instructions. Purification of the indexed products was performed using MagSi-NGS Prep Plus Magnetic beads (Steinbrenner Laborsysteme GmbH, Wiesenbach, Germany) as recommended by the manufacturer, and normalization was performed with the Janus Automated Workstation from Perkin Elmer (Perkin Elmer, Waltham Massachusetts, USA). Sequencing was conducted using Illumina MiSeq platform using dual indexing and MiSeq reagent kit v3 (600 cycles) as recommended by the manufacturer.

**Supplementary Table 1** Full models 1a-1c explaining the offspring’s gut bacteria alpha diversity (from richness estimators). Significant *P* values of explanatory variables are in bold. R^2^ indicates the conditional coefficient of determination. All covariates are log_n_-transformed and then z-transformed (mean = 0, SD = 1) to meet model requirements and increase model interpretability. ^(1)^ coded with “female” as the reference category, ^(2)^ coded with “infant” as the reference category, ^(3)^ coded with “lean” as the reference category, ^(4)^ coded with the group “MOT” as the reference category.

|  | **Full model 1a (ObservedASVs) (R^2^ = 0.34)** | | | | | **Full model 1b (ACE) (R^2^ = 0.41)** | | | | | **Full model 1c (PD) (R^2^ = 0.32)** | | | | |
| --- | --- | --- | --- | --- | --- | --- | --- | --- | --- | --- | --- | --- | --- | --- | --- |
| **Predictors** | **ꞵ** | **SE** | **CI 2.5-97.5%** | **Z** | **P-Value** | **ꞵ** | **SE** | **CI 2.5-97.5%** | **Z** | **P-Value** | **ꞵ** | **SE** | **CI 2.5-97.5%** | **Z** | **P-Value** |
| (Intercept) | 1084.03 | 41.66 | 1002.38 – 1165.68 | 26.02 | **<0.001** | 1397.05 | 53.65 | 1291.90 – 1502.20 | 26.04 | **<0.001** | 69.90 | 2.40 | 65.19 – 74.61 | 29.09 | **<0.001** |
| Early-preGC | -32.44 | 22.38 | -76.30 – 11.42 | -1.45 | 0.147 | -55.59 | 28.82 | -112.07 – 0.89 | -1.93 | 0.054 | -2.79 | 1.29 | -5.32 – -0.26 | -2.16 | **0.031** |
| Late-preGC | 30.90 | 23.17 | -14.52 – 76.32 | 1.33 | 0.182 | 35.93 | 29.84 | -22.56 – 94.41 | 1.20 | 0.229 | 1.00 | 1.34 | -1.62 – 3.62 | 0.75 | 0.453 |
| PostGC | 33.03 | 32.18 | -30.05 – 96.10 | 1.03 | 0.305 | 55.93 | 41.44 | -25.30 – 137.15 | 1.35 | 0.177 | 2.17 | 1.86 | -1.47 – 5.81 | 1.17 | 0.242 |
| OffspringGC | -34.27 | 22.33 | -78.04 – 9.51 | -1.53 | 0.125 | -42.37 | 28.76 | -98.74 – 14.00 | -1.47 | 0.141 | -0.89 | 1.29 | -3.41 – 1.64 | -0.69 | 0.492 |
| ^1^Sex [M] | 23.25 | 23.30 | -22.41 – 68.92 | 1.00 | 0.318 | 38.18 | 30.00 | -20.63 – 96.98 | 1.27 | 0.203 | 0.33 | 1.34 | -2.30 – 2.97 | 0.25 | 0.805 |
| ^2^Age [adu] | 83.07 | 54.64 | -24.02 – 190.16 | 1.52 | 0.128 | 154.95 | 70.36 | 17.05 – 292.86 | 2.20 | **0.028** | 4.79 | 3.15 | -1.39 – 10.96 | 1.52 | 0.129 |
| ^2^Age [juv] | 129.73 | 57.41 | 17.21 – 242.25 | 2.26 | **0.024** | 214.15 | 73.93 | 69.25 – 359.04 | 2.90 | **0.004** | 12.08 | 3.31 | 5.59 – 18.57 | 3.65 | **<0.001** |
| ^3^Season [Rich] | -150.86 | 25.74 | -201.31 – -100.40 | -5.86 | **<0.001** | -231.61 | 33.15 | -296.59 – -166.64 | -6.99 | **<0.001** | -7.60 | 1.48 | -10.51 – -4.69 | -5.12 | **<0.001** |
| ^4^Group [MST] | -115.78 | 34.37 | -183.14 – -48.41 | -3.37 | **0.001** | -140.16 | 44.26 | -226.91 – -53.41 | -3.17 | **0.002** | -4.70 | 1.98 | -8.59 – -0.82 | -2.37 | **0.018** |
| ^4^Group [SST] | -137.74 | 41.22 | -218.52 – -56.95 | -3.34 | **0.001** | -173.22 | 53.08 | -277.25 – -69.19 | -3.26 | **0.001** | -4.89 | 2.38 | -9.55 – -0.23 | -2.06 | **0.040** |
| Early-preGC*Age[adu] | -49.26 | 73.03 | -192.39 – 93.87 | -0.67 | 0.500 | -41.63 | 94.04 | -225.94 – 142.69 | -0.44 | 0.658 | -3.43 | 4.21 | -11.69 – 4.82 | -0.82 | 0.415 |
| Early-preGC*Age[juv] | 60.01 | 45.52 | -29.21 – 149.23 | 1.32 | 0.187 | 95.93 | 58.62 | -18.96 – 210.82 | 1.64 | 0.102 | 3.81 | 2.63 | -1.34 – 8.95 | 1.45 | 0.147 |
| Late-preGC*Age[adu] | -50.00 | 35.96 | -120.48 – 20.48 | -1.39 | 0.164 | -67.92 | 46.31 | -158.69 – 22.84 | -1.47 | 0.142 | -3.32 | 2.07 | -7.39 – 0.74 | -1.60 | 0.109 |
| Late-preGC*Age[juv] | -60.98 | 39.51 | -138.42 – 16.46 | -1.54 | 0.123 | -73.95 | 50.88 | -173.68 – 25.77 | -1.45 | 0.146 | -3.15 | 2.28 | -7.61 – 1.32 | -1.38 | 0.167 |
| PostGC*Age[adu] | 14.92 | 50.31 | -83.69 – 113.54 | 0.30 | 0.767 | 5.51 | 64.79 | -121.48 – 132.49 | 0.09 | 0.932 | 1.22 | 2.90 | -4.47 – 6.91 | 0.42 | 0.675 |
| PostGC*Age[juv] | 9.83 | 39.94 | -68.45 – 88.11 | 0.25 | 0.806 | 1.92 | 51.43 | -98.88 – 102.73 | 0.04 | 0.970 | -1.46 | 2.30 | -5.97 – 3.06 | -0.63 | 0.527 |
| OffspringGC*Age[adu] | 39.06 | 40.58 | -40.47 – 118.60 | 0.96 | 0.336 | 36.21 | 52.25 | -66.21 – 138.63 | 0.69 | 0.488 | 0.65 | 2.34 | -3.94 – 5.24 | 0.28 | 0.781 |
| OffspringGC*Age[juv] | 35.32 | 35.61 | -34.47 – 105.11 | 0.99 | 0.321 | 38.79 | 45.85 | -51.08 – 128.67 | 0.85 | 0.398 | 2.82 | 2.05 | -1.21 – 6.84 | 1.37 | 0.170 |

**Supplementary Table 2** Full model 1d-1e explaining the offspring’s gut bacteria alpha diversity (from richness-evenness estimators). Significant *P* values of explanatory variables are in bold. R^2^ indicates the conditional coefficient of determination. All covariates are log_n_-transformed and then z-transformed (mean = 0, SD = 1) to meet model requirements and increase model interpretability. ^(1)^ coded with “female” as the reference category, ^(2)^ coded with “infant” as the reference category, ^(3)^ coded with “lean” as the reference category, ^(4)^ coded with the group “MOT” as the reference category.

|  | **Full model 1d (Shannon) (R^2^ = 0.18)** | | | | | **Full model 1e (inverse Simpson) (R^2^ = 0.15)** | | | | |
| --- | --- | --- | --- | --- | --- | --- | --- | --- | --- | --- |
| **Predictors** | **ꞵ** | **SE** | **CI 2.5-97.5%** | **Z** | **P-Value** | **ꞵ** | **SE** | **CI 2.5-97.5%** | **Z** | **P-Value** |
| (Intercept) | 5.83 | 0.12 | 5.60 – 6.06 | 49.78 | **<0.001** | 125.34 | 13.13 | 99.60 – 151.08 | 9.54 | **<0.001** |
| Early-preGC | 0.03 | 0.06 | -0.09 – 0.15 | 0.47 | 0.635 | 13.49 | 7.06 | -0.34 – 27.31 | 1.91 | 0.056 |
| Late-preGC | 0.07 | 0.07 | -0.06 – 0.20 | 1.04 | 0.297 | 11.13 | 7.31 | -3.19 – 25.45 | 1.52 | 0.128 |
| PostGC | 0.00 | 0.09 | -0.18 – 0.18 | 0.00 | 0.997 | -8.09 | 10.15 | -27.98 – 11.80 | -0.80 | 0.425 |
| OffspringGC | -0.13 | 0.06 | -0.25 – -0.00 | -2.01 | **0.044** | -15.62 | 7.04 | -29.42 – -1.81 | -2.22 | **0.027** |
| ^1^Sex [M] | 0.00 | 0.07 | -0.13 – 0.13 | 0.04 | 0.967 | -3.82 | 7.35 | -18.22 – 10.58 | -0.52 | 0.603 |
| ^2^Age [adu] | 0.03 | 0.15 | -0.27 – 0.33 | 0.22 | 0.827 | -14.74 | 17.23 | -48.50 – 19.03 | -0.86 | 0.392 |
| ^2^Age [juv] | -0.03 | 0.16 | -0.35 – 0.28 | -0.21 | 0.837 | -47.58 | 18.10 | -83.06 – -12.11 | -2.63 | **0.009** |
| ^3^Season [Rich] | -0.25 | 0.07 | -0.40 – -0.11 | -3.51 | **<0.001** | -3.08 | 8.12 | -18.98 – 12.83 | -0.38 | 0.705 |
| ^4^Group [MST] | -0.29 | 0.10 | -0.48 – -0.10 | -3.03 | **0.002** | -23.76 | 10.84 | -45.00 – -2.52 | -2.19 | **0.028** |
| ^4^Group [SST] | -0.37 | 0.12 | -0.60 – -0.14 | -3.18 | **0.001** | -40.48 | 13.00 | -65.95 – -15.01 | -3.12 | **0.002** |
| Early-preGC*Age[adu] | -0.16 | 0.21 | -0.56 – 0.25 | -0.76 | 0.445 | -30.36 | 23.02 | -75.49 – 14.77 | -1.32 | 0.187 |
| Early-preGC*Age[juv] | 0.02 | 0.13 | -0.23 – 0.27 | 0.16 | 0.872 | -16.29 | 14.35 | -44.42 – 11.84 | -1.14 | 0.256 |
| Late-preGC*Age[adu] | -0.04 | 0.10 | -0.24 – 0.16 | -0.41 | 0.679 | -9.64 | 11.34 | -31.86 – 12.58 | -0.85 | 0.395 |
| Late-preGC*Age[juv] | -0.11 | 0.11 | -0.32 – 0.11 | -0.96 | 0.337 | 10.94 | 12.46 | -13.48 – 35.35 | 0.88 | 0.380 |
| PostGC*Age[adu] | 0.01 | 0.14 | -0.26 – 0.29 | 0.09 | 0.928 | 7.09 | 15.86 | -24.00 – 38.18 | 0.45 | 0.655 |
| PostGC*Age[juv] | 0.13 | 0.11 | -0.09 – 0.35 | 1.17 | 0.241 | 17.95 | 12.59 | -6.73 – 42.63 | 1.43 | 0.154 |
| OffspringGC*Age[adu] | 0.16 | 0.11 | -0.06 – 0.38 | 1.40 | 0.162 | 20.23 | 12.79 | -4.84 – 45.30 | 1.58 | 0.114 |
| OffspringGC*Age[juv] | 0.13 | 0.10 | -0.06 – 0.33 | 1.31 | 0.190 | -2.32 | 11.23 | -24.32 – 19.69 | -0.21 | 0.837 |

**Supplementary Table 3** Reduced models 1a-1c explaining the offspring’s gut bacteria alpha diversity (from richness estimators). Significant *P* values of explanatory variables are in bold. The model was tested via model comparison with null models: Model 1a: χ2 = 72.85, df = 11, p < 0.001; Model 1b: χ2 = 91.98, df = 11, p < 0.001; Model 1c: χ2 = 66.38, df = 11, p < 0.001. R^2^ indicates conditional coefficient of determination. All covariates are log_n_-transformed and then z-transformed (mean = 0, SD = 1) to meet model requirements and increase model interpretability. ^(1)^ coded with “female” as the reference category, ^(2)^ coded with “infant” as the reference category, ^(3)^ coded with “lean” as the reference category, ^(4)^ coded with the group “MOT” as the reference category.

|  | **Model 1a (ObservedASVs) (R^2^ = 0.32)** | | | | | | **Model 1b (ACE) (R^2^ = 0.39)** | | | | | | **Model 1c (PD) (R^2^ = 0.29)** | | | | |
| --- | --- | --- | --- | --- | --- | --- | --- | --- | --- | --- | --- | --- | --- | --- | --- | --- | --- |
| **Predictors** | **ꞵ** | **SE** | **CI 2.5-97.5%** | **Z** | **P-Value** | **ꞵ** | | **SE** | **CI 2.5-97.5%** | **Z** | **P-Value** | **ꞵ** | | **SE** | **CI 2.5-97.5%** | **Z** | **P-Value** |
| (Intercept) | 1046.47 | 34.87 | 978.14 – 1114.81 | 30.01 | **-** | 1350.98 | | 45.03 | 1262.72 – 1439.23 | 30.00 | **-** | 68.21 | | 2.01 | 64.26 – 72.15 | 33.88 | **-** |
| Early-preGC | -27.84 | 15.28 | -57.79 – 2.11 | -1.82 | 0.068 | -40.47 | | 19.73 | -79.15 – -1.80 | -2.05 | **0.040** | -2.09 | | 0.88 | -3.82 – -0.36 | -2.37 | **0.018** |
| Late- preGC | 9.87 | 14.75 | -19.03 – 38.78 | 0.67 | 0.503 | 8.11 | | 19.05 | -29.22 – 45.44 | 0.43 | 0.670 | 0.00 | | 0.85 | -1.67 – 1.67 | 0.00 | 0.999 |
| PostGC | 25.31 | 14.89 | -3.86 – 54.49 | 1.70 | 0.089 | 36.99 | | 19.22 | -0.69 – 74.67 | 1.92 | 0.054 | 1.24 | | 0.86 | -0.44 – 2.93 | 1.45 | 0.148 |
| OffspringGC | -11.36 | 13.36 | -37.54 – 14.82 | -0.85 | 0.395 | -17.42 | | 17.25 | -51.24 – 16.39 | -1.01 | 0.313 | -0.01 | | 0.77 | -1.52 – 1.50 | -0.02 | 0.987 |
| ^1^Sex [M] | 19.01 | 22.46 | -25.02 – 63.03 | 0.85 | 0.397 | 33.29 | | 29.01 | -23.57 – 90.14 | 1.15 | 0.251 | 0.20 | | 1.30 | -2.34 – 2.74 | 0.15 | 0.879 |
| ^2^Age [adu] | 137.55 | 32.13 | 74.58 – 200.52 | 4.28 | **<0.001** | 220.67 | | 41.49 | 139.34 – 301.99 | 5.32 | **<0.001** | 8.62 | | 1.86 | 4.99 – 12.26 | 4.65 | **<0.001** |
| ^2^Age [juv] | 150.26 | 40.44 | 71.00 – 229.51 | 3.72 | **<0.001** | 251.13 | | 52.22 | 148.78 – 353.49 | 4.81 | **<0.001** | 11.66 | | 2.33 | 7.08 – 16.23 | 4.99 | **<0.001** |
| ^3^Season [Rich] | -145.82 | 25.18 | -195.17 – -96.47 | -5.79 | **<0.001** | -225.83 | | 32.52 | -289.56 – -162.10 | -6.95 | **<0.001** | -7.00 | | 1.45 | -9.85 – -4.15 | -4.82 | **<0.001** |
| ^4^Group [MST] | -85.33 | 28.34 | -140.89 – -29.78 | -3.01 | **0.003** | -101.66 | | 36.60 | -173.40 – -29.91 | -2.78 | **0.005** | -3.11 | | 1.64 | -6.32 – 0.10 | -1.90 | 0.058 |
| ^4^Group [SST] | -114.88 | 32.73 | -179.02 – -50.73 | -3.51 | **<0.001** | -147.98 | | 42.27 | -230.82 – -65.14 | -3.50 | **<0.001** | -4.51 | | 1.89 | -8.21 – -0.81 | -2.39 | **0.017** |

**Supplementary Table 4** Reduced model 1d and 1e explaining the offspring’s gut bacteria alpha diversity (from richness-evenness estimators). Significant *P* values of explanatory variables are in bold. The model was tested via model comparison with null models: Model 1d: χ2 = 29.26, df = 15, p = 0.015; Model 1e: χ2 = 20.08, df = 11, p = 0.044. R^2^ indicates the conditional coefficient of determination. All covariates represent average values log-transformed and then z-transformed. All covariates are log_n_-transformed and then z-transformed (mean = 0, SD = 1) to meet model requirements and increase model interpretability. ^(1)^ coded with “female” as the reference category, ^(2)^ coded with “infant” as the reference category, ^(3)^ coded with “lean” as the reference category, ^(4)^ coded with the group “MOT” as the reference category.

|  | **Model 1d (Shannon) (R^2^ = 0.14)** | | | | | **Model 1e (inverse Simpson) (R^2^ = 0.10)** | | | | |
| --- | --- | --- | --- | --- | --- | --- | --- | --- | --- | --- |
| **Predictors** | **ꞵ** | **SE** | **CI 2.5-97.5%** | **Z** | **P-Value** | **ꞵ** | **SE** | **CI 2.5-97.5%** | **Z** | **P-Value** |
| (Intercept) | 5.71 | 0.10 | 5.51 – 5.90 | 57.83 | **-** | 118.64 | 11.93 | 95.27 – 142.02 | 9.95 | **-** |
| Early-preGC | -0.01 | 0.04 | -0.10 – 0.08 | -0.22 | 0.828 | 7.22 | 5.52 | -3.61 – 18.05 | 1.31 | 0.191 |
| Late- preGC | 0.04 | 0.04 | -0.04 – 0.12 | 0.92 | 0.358 | 9.73 | 5.13 | -0.34 – 19.79 | 1.89 | 0.058 |
| PostGC | 0.02 | 0.04 | -0.07 – 0.10 | 0.36 | 0.716 | -6.54 | 5.32 | -16.97 – 3.88 | -1.23 | 0.218 |
| OffspringGC | -0.03 | 0.04 | -0.11– 0.05 | -0.75 | 0.455 | -7.82 | 4.46 | -16.55 – 0.92 | -1.75 | 0.080 |
| ^1^Sex [M] | 0.00 | 0.07 | -0.13 – 0.13 | -0.01 | 0.992 | -6.16 | 7.72 | -21.30 – 8.98 | -0.80 | 0.425 |
| ^2^Age [adu] | 0.14 | 0.09 | -0.05 – 0.32 | 1.45 | 0.148 | 2.70 | 10.97 | -18.81 – 24.20 | 0.25 | 0.806 |
| ^2^Age [juv] | 0.02 | 0.12 | -0.22 – 0.25 | 0.09 | 0.925 | -28.27 | 13.82 | -55.36 – -1.19 | -2.05 | **0.041** |
| ^3^Season [Rich] | -0.24 | 0.07 | -0.38 – -0.10 | -3.37 | **0.001** | -5.12 | 7.89 | -20.58 – 10.35 | -0.65 | 0.517 |
| ^4^Group [MST] | -0.22 | 0.08 | -0.39 – -0.06 | -2.71 | **0.007** | -21.75 | 9.80 | -40.97 – -2.54 | -2.22 | **0.026** |
| ^4^Group [SST] | -0.27 | 0.10 | -0.45 – -0.08 | -2.76 | **0.006** | -26.49 | 11.42 | -48.88 – -4.09 | -2.32 | **0.020** |

**Supplementary Table 5** Full linear models explaining the *Firmicutes* to *Bacteroidota* ratio of offspring’s gut bacteria during the lean (Full-Model L) and the rich season (Full-Model R). ^(1)^ indicates that the predictor has been log_n_-transformed and then z-transformed with mean = 0 and SD = 1 to meet model requirements and to increase model interpretability; ^(2)^ indicates that the predictor has been z-transformed with mean = 0 and SD = 1; ^(3)^ coded with the group “MOT” as the reference category; ^(3)^ coded with “female” as the reference category.

| **Predictor** | **Full-Model L (pseudo-R^2^ = 0.32)** | | | | | **Full-Model R (pseudo-R^2^ = 0.91)** | | | | |
| --- | --- | --- | --- | --- | --- | --- | --- | --- | --- | --- |
|  | **Estimate** | **SE** | **95% C.I.** | **LRT** | **P-value** | **Estimate** | **SE** | **95% C.I.** | **LRT** | **P-value** |
| Intercept | 1.123 | 0.006 | 1.112 – 1.134 | - | - | 1.170 | 0.007 | 1.156 – 1.183 | - | - |
| Early-preGC^1^ | -0.004 | 0.005 | -0.015 – 0.006 | - | - | -0.016 | 0.008 | -0.031 – 0.000 | - | - |
| Late-preGC^1^ | -0.002 | 0.004 | -0.010 – 0.006 | - | - | -0.018 | 0.006 | -0.029 – -0.007 | - | - |
| PostGC^1^ | -0.005 | 0.005 | -0.015 – 0.005 | - | - | 0.008 | 0.008 | -0.007 – 0.023 | - | - |
| OffspringGC^1^ | -0.007 | 0.004 | -0.016 – 0.002 | - | - | 0.035 | 0.007 | 0.021 – 0.049 | - | - |
| Age^2^ | -0.001 | 0.005 | -0.010 – 0.008 | - | - | -0.042 | 0.011 | -0.064 – -0.020 | - | - |
| Sex[M]^3^ | 0.003 | 0.007 | -0.010 – 0.017 | 0.205 | 0.651 | 0.003 | 0.008 | -0.013 – 0.019 | 0.157 | 0.692 |
| ^1^Early-preGC*Age^2^ | -0.011 | 0.006 | -0.023 – 0.001 | 2.859 | 0.091 | -0.024 | 0.007 | -0.039 – -0.010 | 8.333 | **0.004** |
| ^1^Late-preGC*Age^2^ | 0.007 | 0.005 | -0.003 – 0.016 | 1.849 | 0.174 | 0.014 | 0.009 | -0.003 – 0.031 | 2.275 | 0.131 |
| ^1^PostGC*Age^2^ | 0.007 | 0.005 | -0.002 – 0.016 | 2.304 | 0.129 | 0.031 | 0.008 | 0.016 – 0.047 | 10.725 | **0.001** |
| ^1^OffspringGC*Age^2^ | 0.001 | 0.006 | -0.010 – 0.012 | 0.029 | 0.865 | 0.017 | 0.007 | 0.003 – 0.032 | 5.034 | **0.025** |

**Supplementary Table 6** Effect of *Early-preGC* in interaction with age on gut bacterial composition during the rich season. Infants are excluded from the analyses. UN = unclassified at the genus level.

| **Taxon** | **Early-preGC effect at age = mean** | **Early-preGC effect at age = mean+1SD** | **Early-preGC effect at age = mean-1SD** |
| --- | --- | --- | --- |
| [Bacteroides] pectinophilus group | 0.824 | 1.208 | 0.441 |
| [Clostridium] methylpentosum group (UN) | 0.000 | -0.544 | 0.544 |
| [Clostridium] methylpentosum group (UN) | 0.000 | 0.329 | -0.329 |
| [Eubacterium] coprostanoligenes group (UN) | 1.503 | 2.501 | 0.504 |
| [Eubacterium] coprostanoligenes group (UN) | 0.000 | 0.658 | -0.658 |
| [Eubacterium] coprostanoligenes group (UN) | -0.533 | -1.009 | -0.056 |
| [Eubacterium] ventriosum group | 0.000 | -0.607 | 0.607 |
| [Ruminococcus] gauvreauii group | 0.000 | -0.458 | 0.458 |
| Acetitomaculum | 1.643 | 2.287 | 1.000 |
| Acetivibrio | -1.689 | -3.042 | -0.336 |
| Actinomycetaceae (UN) | 0.288 | 0.577 | -0.001 |
| Alloprevotella | 0.810 | 1.522 | 0.099 |
| Alloscardovia | 0.520 | 0.858 | 0.183 |
| Anaerobium | 0.000 | 0.544 | -0.544 |
| Anaerofustis | 0.351 | 0.629 | 0.074 |
| Anaerostipes | 0.324 | 0.669 | -0.021 |
| Anaerovoracaceae (UN) | -0.559 | -1.150 | 0.031 |
| Anaplasmataceae (UN) | 0.257 | 0.473 | 0.041 |
| Angelakisella | 0.000 | 0.483 | -0.483 |
| Bacilli RF39 (UN) | 0.263 | 0.507 | 0.020 |
| Bacilli RF39 (UN) | -0.457 | -0.800 | -0.114 |
| Bacteroidales (UN) | -1.616 | -3.820 | 0.588 |
| Bacteroidales RF16 group (UN) | -0.846 | -1.869 | 0.177 |
| Beijerinckiaceae (UN) | 0.270 | 0.447 | 0.093 |
| Bifidobacterium | -3.328 | -5.936 | -0.720 |
| Blautia | 0.457 | 0.946 | -0.032 |
| Brachyspira | 0.460 | 1.164 | -0.245 |
| CAG-352 | 1.019 | 2.456 | -0.418 |
| Campylobacter | 0.620 | 1.165 | 0.075 |
| Candidatus Rhabdochlamydia | 0.226 | 0.425 | 0.027 |
| Carnobacteriaceae (UN) | 0.367 | 0.658 | 0.076 |
| Cellulomonadaceae (UN) | 0.270 | 0.447 | 0.093 |
| Cellulosilyticum | 0.000 | 0.425 | -0.425 |
| Christensenellaceae R-7 group | -0.788 | -1.396 | -0.180 |
| Clostridia UCG-014 (UN) | 1.819 | 2.810 | 0.829 |
| Clostridia UCG-014 (UN) | 0.785 | 1.354 | 0.217 |
| Clostridia UCG-014 (UN) | 0.213 | 0.438 | -0.012 |
| Clostridia UCG-014 (UN) | -0.243 | -0.371 | -0.115 |
| Clostridia vadinBB60 group (UN) | 0.257 | 0.473 | 0.041 |
| Clostridia vadinBB60 group (UN) | 0.234 | 0.498 | -0.029 |
| Clostridia vadinBB60 group (UN) | 0.000 | 1.013 | -1.013 |
| Clostridia vadinBB60 group (UN) | -0.467 | -0.947 | 0.013 |
| Clostridia vadinBB60 group (UN) | -0.530 | -1.126 | 0.067 |
| Clostridia vadinBB60 group (UN) | -0.543 | -0.864 | -0.223 |
| Clostridium sensu stricto 5 | -1.808 | -4.009 | 0.393 |
| Comamonadaceae (UN) | -0.684 | -1.195 | -0.173 |
| Coprococcus | 1.467 | 2.958 | -0.023 |
| Coriobacteriales (UN) | 0.499 | 0.813 | 0.184 |
| Corynebacterium | 0.257 | 0.473 | 0.041 |
| D05-2 (UN) | 0.228 | 0.463 | -0.008 |
| Desulfovibrio | 0.000 | -0.713 | 0.713 |
| Devosia | 0.259 | 0.428 | 0.090 |
| Devosiaceae (UN) | 0.276 | 0.552 | 0.000 |
| dgA-11 gut group | 0.000 | -1.066 | 1.066 |
| Dorea | 0.680 | 1.192 | 0.168 |
| Eggerthellaceae (UN) | 0.555 | 0.802 | 0.307 |
| EMP-G18 | -1.913 | -3.644 | -0.182 |
| Enterobacteriaceae (UN) | 1.335 | 2.458 | 0.212 |
| Enterococcaceae (UN) | 1.570 | 2.725 | 0.415 |
| Erysipelatoclostridiaceae (UN) | 0.595 | 1.550 | -0.359 |
| Erysipelotrichaceae UCG-006 | 0.388 | 0.755 | 0.021 |
| Faecalibacterium | 0.629 | 0.987 | 0.270 |
| Fusobacterium | 0.251 | 0.400 | 0.102 |
| Gammaproteobacteria Incertae Sedis | 0.000 | 0.395 | -0.395 |
| Gemella | 0.681 | 1.071 | 0.291 |
| gut metagenome | 0.430 | 0.976 | -0.117 |
| Helicobacteraceae (UN) | 0.214 | 0.610 | -0.181 |
| Incertae Sedis | 0.000 | -0.337 | 0.337 |
| Intestinimonas | 0.000 | -0.540 | 0.540 |
| Izemoplasmatales (UN) | 0.200 | 0.477 | -0.076 |
| Lachnospira | 0.334 | -0.085 | 0.753 |
| Lachnospiraceae (UN) | 0.454 | 0.997 | -0.089 |
| Lachnospiraceae UCG-001 | 0.991 | 1.760 | 0.222 |
| Lachnospiraceae UCG-007 | 0.449 | 0.859 | 0.039 |
| Lachnospiraceae UCG-009 | 0.316 | 0.540 | 0.092 |
| Lactobacillus | 0.000 | 0.925 | -0.925 |
| Megasphaera | 0.000 | 1.404 | -1.404 |
| Microbacteriaceae (UN) | 0.218 | 0.431 | 0.004 |
| Micromonosporaceae (UN) | 0.350 | 0.661 | 0.039 |
| Mobilitalea | 0.000 | 0.290 | -0.290 |
| Muribaculaceae (UN) | -1.013 | -1.344 | -0.681 |
| Mycobacteriaceae (UN) | 0.365 | 0.643 | 0.087 |
| Negativibacillus | 0.305 | 0.562 | 0.049 |
| Neisseria | 0.348 | 0.735 | -0.038 |
| Oscillibacter | 0.366 | 0.657 | 0.074 |
| Oscillospira | -1.172 | -2.648 | 0.303 |
| Oxalobacter | 0.000 | -0.490 | 0.490 |
| Oxalobacteraceae (UN) | 0.257 | 0.473 | 0.041 |
| Paracaedibacteraceae (UN) | 0.282 | 0.426 | 0.137 |
| Peptococcus | 0.000 | 0.316 | -0.316 |
| Peptostreptococcaceae (UN) | -0.778 | -1.431 | -0.125 |
| Porphyromonas | 0.228 | 0.463 | -0.008 |
| Prevotella 9 | 0.339 | 0.691 | -0.013 |
| Prevotellaceae UCG-003 | 0.832 | 1.995 | -0.331 |
| Proteobacterium (UN) | 0.000 | -0.857 | 0.857 |
| Pseudomonadaceae (UN) | 0.256 | 0.482 | 0.030 |
| RF39 (UN) | -0.782 | -1.380 | -0.183 |
| Rhizobiaceae (UN) | 0.000 | 0.247 | -0.247 |
| Rhodospirillales (UN) | 0.446 | 0.919 | -0.027 |
| Rickettsiella | 0.263 | 0.507 | 0.020 |
| Rikenellaceae (UN) | -0.400 | -1.364 | 0.563 |
| Rodentibacter | 0.451 | 0.767 | 0.135 |
| Roseburia | 0.000 | 0.434 | -0.434 |
| Rothia | 0.373 | 0.748 | -0.001 |
| Ruminococcaceae (UN) | 0.298 | 0.632 | -0.035 |
| Ruminococcaceae (UN) | -0.711 | -1.277 | -0.146 |
| Ruminococcus | 0.000 | 0.438 | -0.438 |
| Sphingomonadaceae (UN) | 0.325 | 0.606 | 0.043 |
| Staphylococcaceae (UN) | 0.192 | 0.501 | -0.118 |
| Streptococcaceae (UN) | 1.622 | 3.256 | -0.013 |
| Tsukamurellaceae (UN) | 0.319 | 0.536 | 0.101 |
| Tyzzerella | 1.928 | 3.586 | 0.270 |
| UCG-002 (UN) | -0.597 | -1.207 | 0.012 |
| UCG-004 (UN) | -0.590 | -1.587 | 0.407 |
| UCG-009 (UN) | -0.463 | -1.030 | 0.104 |
| UCG-010 (UN) | 0.282 | 0.426 | 0.137 |
| UCG-011 (UN) | 0.000 | -0.654 | 0.654 |
| Bacteroidaceae (UN) | 1.196 | 2.234 | 0.159 |
| Ureaplasma | 0.628 | 1.292 | -0.037 |
| Yersiniaceae (UN) | 0.263 | 0.507 | 0.020 |


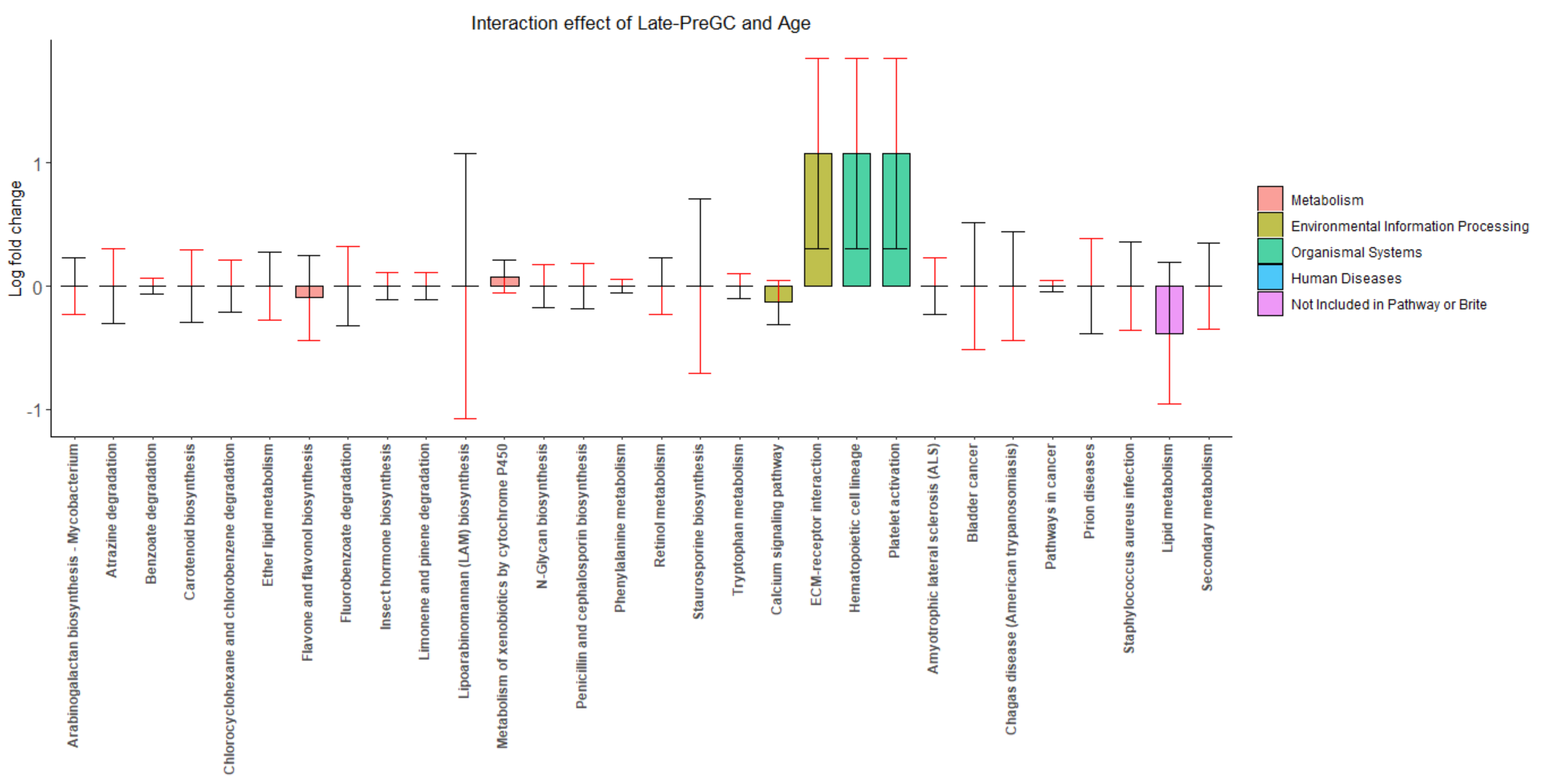


**Supplementary Figure 1 –** Predicted functional pathways significantly affected by the prenatal maternal GCs and offspring age (*Late-PreGC*Age*). KOs are grouped at level 3 of KEGG database. Only significantly affected pathways are plotted (BH adjusted p-values). Colored bars indicate the effect of an increase in 1SD of *Late-preGC* (SD = 67.0 ng/g) estimated at the mean value of age (mean = 4.7 years), and at the mean value of all the other predictors. Red and black lines indicate the effect estimated at the mean plus1SD (red = 7.1 years) and at the mean minus1SD (black = 2.3) of age. When the bar is not plotted, the effect of the same increase in maternal GCs at the mean age is 0. Taxa showing the black head (estimates at 2.3 years) further from 0 than the red head (estimates at 7.1 years) show an effect that is reduced with age.


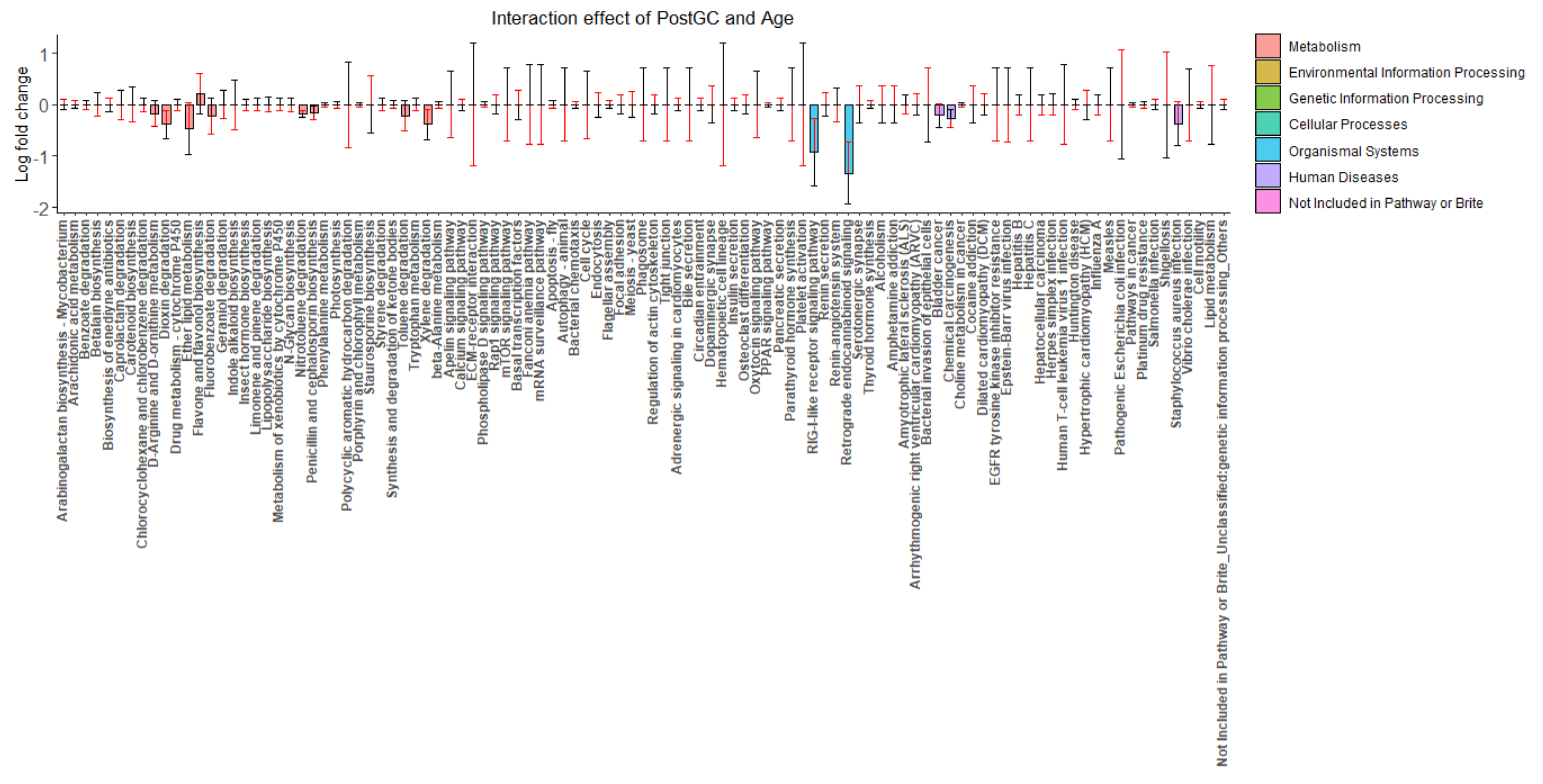


**Supplementary Figure 2 –** Predicted functional pathways significantly affected by the postnatal maternal GCs and offspring age (*PostGC*Age*). KOs are grouped at level 3 of KEGG database. Only significantly affected pathways are plotted (BH adjusted p-values). Colored bars indicate the effect of an increase in 1SD of *PostGC* (SD = 52.7 ng/g) estimated at the mean value of age (mean = 4.7 years), and at the mean value of all the other predictors. Red and black lines indicate the effect estimated at the mean plus1SD (red = 7.1 years) and at the mean minus1SD (black = 2.3) of age. When the bar is not plotted, the effect of the same increase in maternal GCs at the mean age is 0. Taxa showing the black head (estimates at 2.3 years) further from 0 than the red head (estimates at 7.1 years) show an effect that is reduced with age.


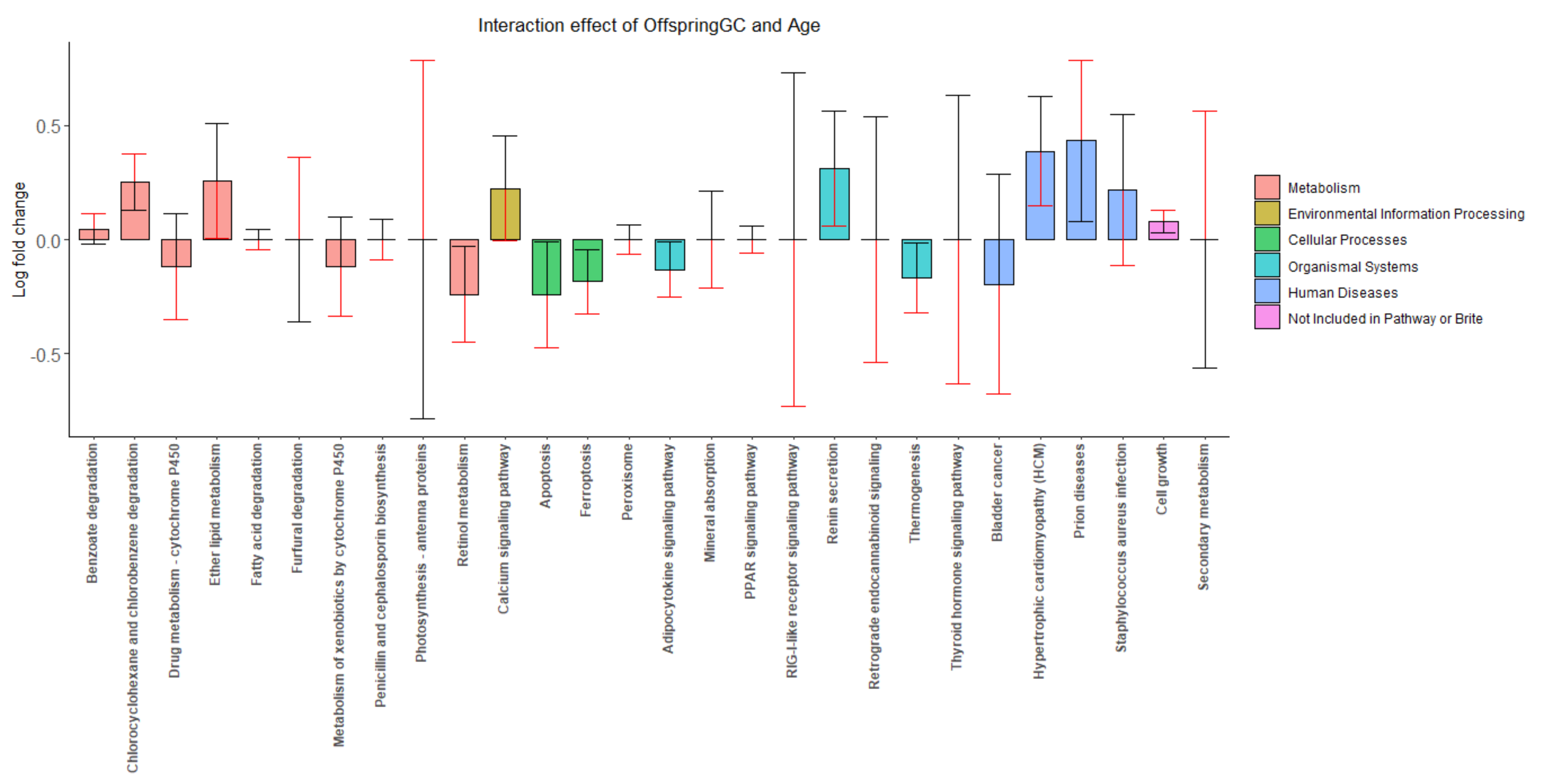


**Supplementary Figure 3 –** Predicted functional pathways significantly affected by the offspring GCs and offspring age (*OffspringGC*Age*). KOs are grouped at level 3 of KEGG database. Only significantly affected pathways are plotted (BH adjusted p-values). Colored bars indicate the effect of an increase in 1SD of *OffspringGC* (SD = 130.1 ng/g) estimated at the mean value of age (mean = 4.7 years), and at the mean value of all the other predictors. Red and black lines indicate the effect estimated at the mean plus1SD (red = 7.1 years) and at the mean minus1SD (black = 2.3) of age. When the bar is not plotted, the effect of the same increase in maternal GCs at the mean age is 0. Taxa showing the black head (estimates at 2.3 years) further from 0 than the red head (estimates at 7.1 years) show an effect that is reduced with age.
